# Genotype-phenotype correlations in PSACH/EDM1 patients with *COMP* gene variants: a comprehensive review of 830 cases

**DOI:** 10.3389/fendo.2026.1740770

**Published:** 2026-02-19

**Authors:** Xiaolin Ni, Liya Wei, Weibo Xia, Di Wu

**Affiliations:** 1 Department of Endocrinology, Genetics and Metabolism, Beijing Children’s Hospital, Capital Medical University, National Center for Children’s Health, Beijing, China; 2 Department of Endocrinology, Key Laboratory of Endocrinology, National Commission of Health, State Key Laboratory of Complex Severe and Rare Diseases, Peking Union Medical College Hospital, Chinese Academy of Medical Sciences, Beijing, China

**Keywords:** COMP gene, genotype-phenotype correlations, multiple epiphyseal dysplasia-1, pseudoachondroplasia, variant

## Abstract

**Background:**

Pseudoachondroplasia (PSACH) and multiple epiphyseal dysplasia-1 (EDM1) are two rare skeletal diseases that represent distinct endpoints of a continuous phenotypic spectrum with substantial clinical overlap, caused by variants in the gene coding cartilage oligomeric matrix protein (COMP).

**Objectives:**

To summarize the clinical characteristics of PSACH/EDM1 and variants of *COMP* gene, as well as to explore the correlations between them.

**Methods:**

PubMed, China National Knowledge Infrastructure, and Wanfang were searched for case reports and case series of patients with genetic diagnosis of PSACH/EDM1 from the inception to 24 March 2025. The clinical characteristics and gene variants of enrolled patients were analyzed and compared to explore genotype-phenotype correlation.

**Results:**

A total of 830 PSACH/EDM1 patients (471probands) harboring 224 different variants of *COMP* gene were enrolled from 106 articles, with missense variants accounting for the majority (80.8%). Exon 13 (183 probands, 38.9%) and type III (T3) repeat domain (413 probands, 87.7%) were the most commonly affected regions, with c.1417_1419del (p.Asp473del) being the most common hotspot variant. Compared with EDM1, PSACH manifested earlier age of onset (*p* < 0.001), shorter stature (*p* < 0.001), higher rates of lower limb deformity (*p* < 0.001), joint laxity (*p* = 0.041), anterior beaking of the vertebra and irregular/flared metaphysis (*p* < 0.001), while lower rate of joint pain/osteoarthritis (*p* < 0.001) and abnormal femoral head (*p* = 0.008). Missense variants in T3–4 and T3–5 were more likely to cause EDM1 (all *p* < 0.001), while those in T3–1 and T3–6 to T3–8 were associated with a greater frequency of PSACH (*p* = 0.002 to 0.023). Majority of in-frame variants were found in PSACH, as c.1417_1419del (p.Asp473del) being PSACH specific.

**Conclusions:**

PSACH exhibits more severe phenotypes than EDM1, even with phenotypic overlap. In-frame variants are more strongly associated with PSACH, as the hotspot variant p.Asp473del exclusively identified in PSACH. In contrast, missense variants in T3–4 and T3–5 show a stronger association with EDM1.

## Introduction

Mutations in the gene encoding cartilage oligomeric matrix protein (COMP) were identified in three diseases, including pseudoachondroplasia (PSACH; OMIM 177170), multiple epiphyseal dysplasia-1 (EDM1; OMIM 132400), and carpal tunnel syndrome (CTS; OMIM 619161) (1–3). Both PSACH and EDM1 are osteochondrodysplasia inherited in an autosomal dominant manner. However, PSACH generally causes more severe short stature than EDM1, showing characteristic anterior beaking of the vertebral bodies on lateral X-rays in childhood (4, 5). Despite the differences in phenotypic severity, PSACH and EDM1 are not mutually exclusive entities but rather part of a continuous phenotypic spectrum driven by overlapping clinical manifestations that vary in frequency and severity and shared causative variants that can lead to either phenotype.

**Figure 1:**
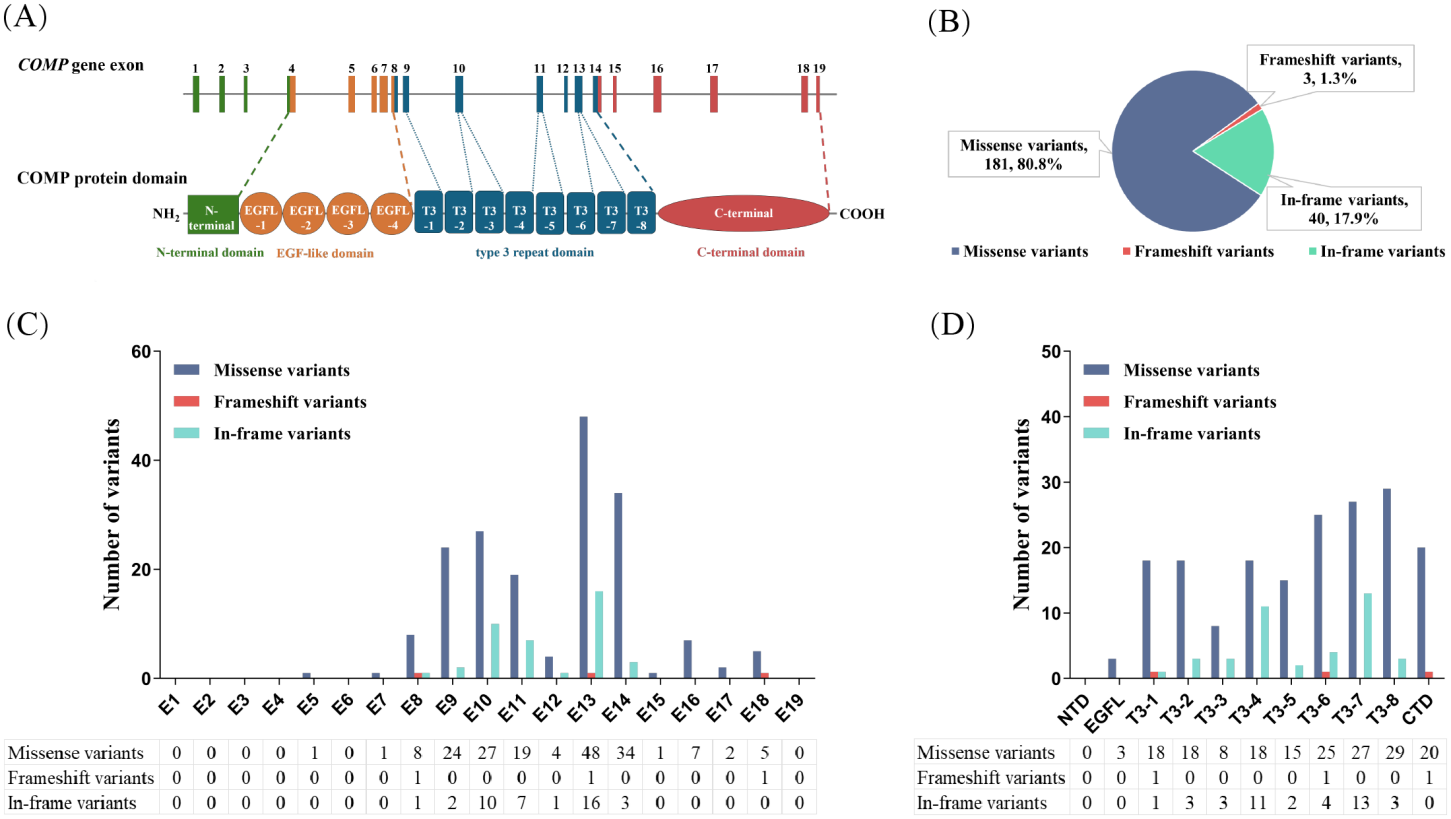
Schematic representation of *COMP* gene, COMP protein, and variants. **(A)** Human *COMP* gene is long of 8533bp, located in chromosome 19 and composed of 19 exons. It encodes a pentameric protein in which the monomer consists of an N-terminal domain (green), four type II epidermal growth factor-like repeats (orange), eight type III calcium-binding repeats (blue), and a C-terminal domain (red). **(B)** Number and percentage of different variant types among 224 variants. **(C)** Distribution of different types of 224 variants over exons 1–19 of *COMP* gene. **(D)** Distribution of different types of 224 variants over domains of COMP protein. COMP, cartilage oligomeric matrix protein; NTD, N-terminal domain; EGFL, type II epidermal growth factor-like repeats; T3, type III calmodulin-like calcium-binding repeats; CTD, C-terminal domain.

COMP is a homopentameric glycoprotein belonging to the family of thrombospondins (TSPs), also known as TSP-5, which is mainly expressed in the extracellular matrix of cartilage, ligament, tendon, and skeletal muscle (6). As shown in **Figure 1A**, each monomeric arm consists of an N-terminal domain (NTD), four type II (T2) epidermal growth factor-like repeats, eight type III (T3) calmodulin-like calcium-binding repeats and a globular C-terminal domain (CTD) (7). Both T3 repeats and CTD bind calcium ions which are critical to correct protein folding. T3 repeats contain five highly conserved aspartic acid residues in both N-type and C-type motifs, with two calcium-binding sites per motif. CTD is a lectin-like-β sandwich domain composed of 15 antiparallel β-strands and containing calcium binding sites. Most variants (99%) in *COMP* gene leading to PSACH/EDM1 occurred in T3 repeat and CTD (8). Notably, in several instances, the same pathogenic variant was reported to cause both PSACH and EDM1.

The current strategy for diagnosis of inherited diseases is to combine clinical characteristics, imaging findings and molecular genetic testing. With the increasing application of gene sequencing, the diagnostic rate of PSACH/EDM1 has been improved. Nevertheless, the overlap in both clinical phenotypic spectra and mutational profiles between PSACH and EDM1 complicates differential diagnosis. Thus, this study aimed to review all published data on PSACH/EDM1 patients up to 24 March 2025, including the *COMP* gene variants, clinical symptoms, radiographic features, and consequently to explore the distribution characteristics of *COMP* gene variants along with the potential genotype-phenotype correlation.

## Methods

### Data sources

PubMed, China National Knowledge Infrastructure (CNKI), and Wanfang were searched from the inception to 24 March 2025. The complete search string used was as follows: (“Cartilage Oligomeric Matrix Protein”[Mesh] OR “cartilage oligomeric matrix protein”[tiab] OR COMP[tiab]) AND (mutation[Mesh] OR mutation[tiab] OR mutations[tiab] OR variant[tiab] OR variants[tiab]) AND (“1900/01/01”[Date - Publication]: “2025/03/24”[Date - Publication]).

### Study selection

**Figure 2:**
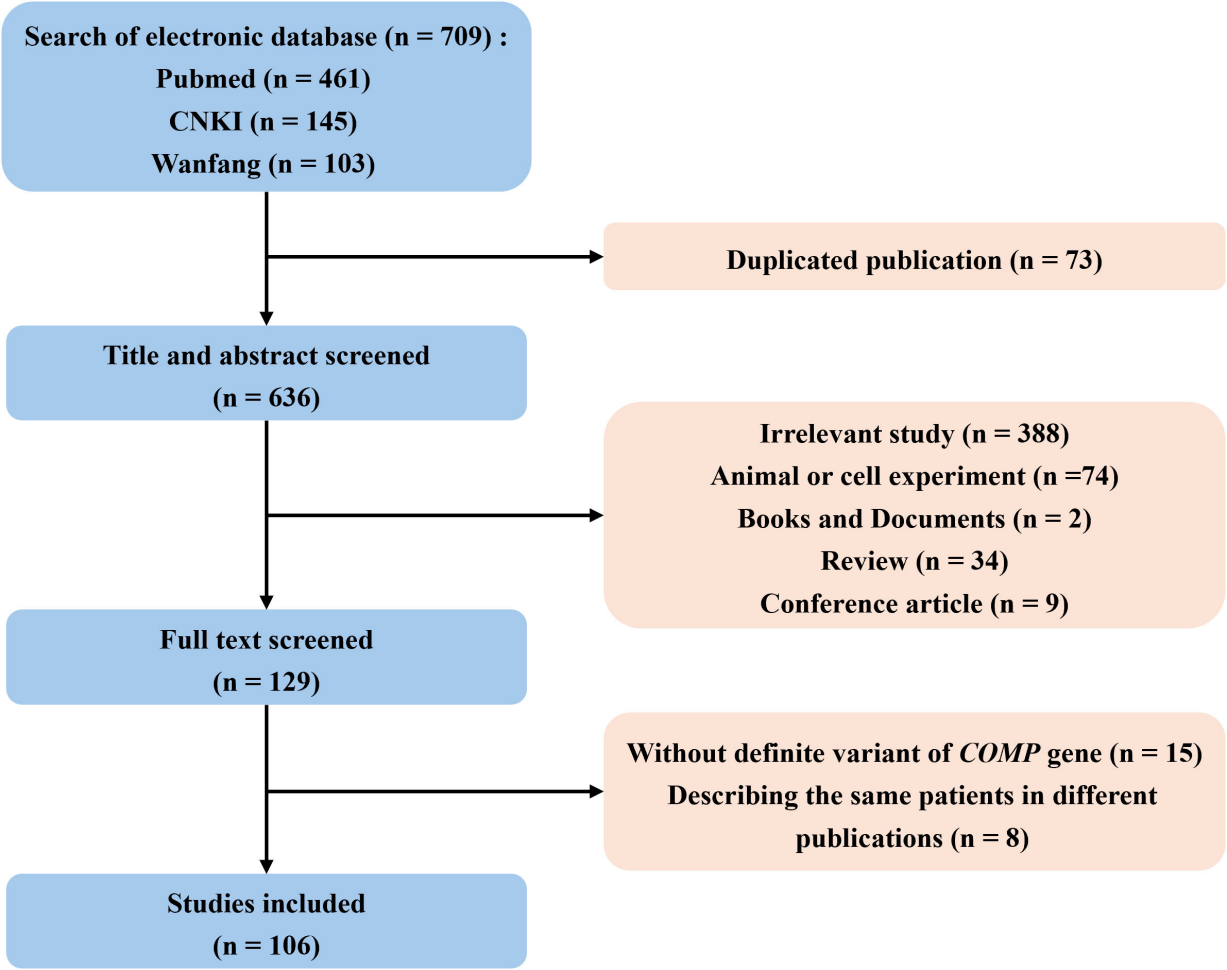
Flow chart for literature inclusion and exclusion. COMP, cartilage oligomeric matrix protein.

Two reviewers screened all articles independently. Eligible studies met the following criteria (1): published in English or Chinese (2); the patients enrolled had genetic diagnosis of ACH or EDM1 (3); the patients enrolled were postnatal. Studies reporting the same patients as other published research will be excluded, and only the study with the most comprehensive description will be retained. Flow chart for literature search is shown in **Figure 2**.

### Data extraction

Variants described only at the amino acid level were converted to variations of nucleotide according to the code. Based on the cDNA reference sequence of *COMP* (NM_000095.3), errors attributed to the wrong numbers of nucleotide sequences were corrected for consistency.

Two investigators reviewed all cases independently. The following information were collected (1): gender (2); age at the visit (3); onset age (4); height or Z score of height (5); clinical symptoms including gait abnormality, lower limb deformity, joint laxity, joint pain, and osteoarthritis (6); radiographic features including changes in epiphysis and metaphysis, dysplasia of femoral head and acetabulum, and anterior beaking of the vertebra (7); variants of nucleotide and amino acid of COMP. Due to varying degrees of data completeness across the included cases, all summaries explicitly report the sample size (n) used for each specific analysis.

### Statistical analyses

Continuous variables were presented as mean ± SD or median (interquartile range) according to the normality of the variables determined by the Shapiro-Wilk test, whereas categorical variables were presented as frequency (percentage). Differences between two groups were analyzed using the independent samples t test for normally distributed continuous data, the Mann-Whitney U test for nonparametric continuous data, and the chi-square test for categorical variables. A two-sided *p* value<0.05 was considered statistically significant. All statistical analyses were performed using IBM SPSS software (version 23, SPSS Inc., Chicago, IL, USA).

## Results

### Genetic characteristics of patients with PSACH/EDM1

A total of 830 patients (471probands) with molecular diagnosis of PSACH/EDM1 caused by variants in *COMP* gene were enrolled from 78 articles published in English (**Supplemental File 1**) and 28 articles published in Chinese, including 474 patients with PSACH and 356 patients with EDM1. Among the 224 variants identified, we found 181 missense variants (80.8%), 40 in-frame variants (17.9%), 3 frameshift variants (1.3%) (**Figure 1B**). Identified 224 variants were distributed across exon 7 to 18, as well as exon 5 (**Figure 1C**). The mutational landscape showed exon 13 was the most frequently affected (n = 65), with exon 10 and exon 14 ranking second (n = 37). Both missense variants and in-frame variants were more frequent in exon 13 than those in other exons. Three variants (1.3%) were identified in the EGF-like domain, 200 variants (89.3%) in the T3 repeat domain, and 21 variants (9.4%) in the CTD (**Figure 1D**).

**Figure 3:**
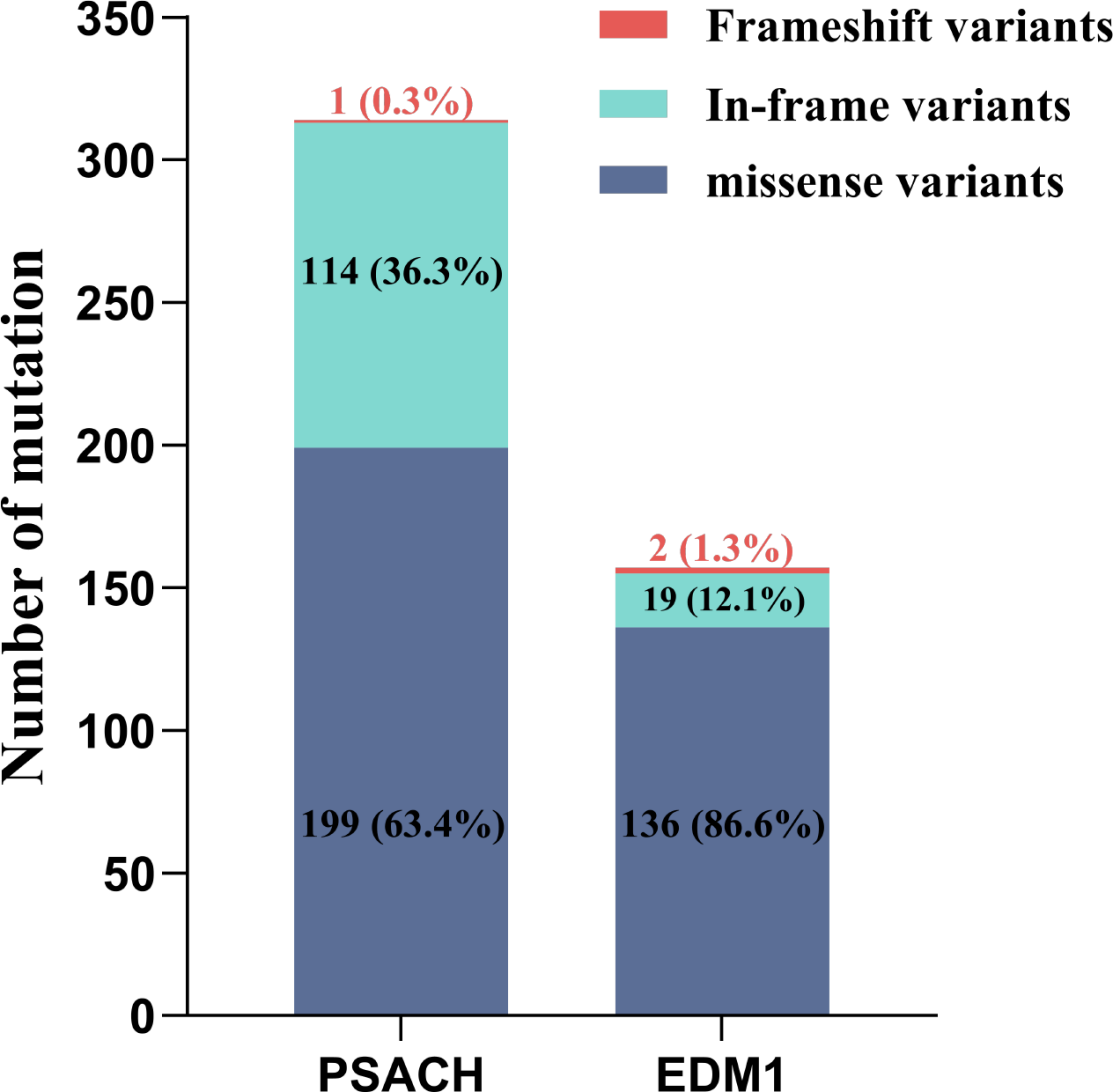
Number and percentage of different variant types in *COMP* gene in 314 PSACH probands and 157 EDM1 probands. COMP, cartilage oligomeric matrix protein; PSACH, pseudoachondroplasia; EDM1, multiple epiphyseal dysplasia-1.

**Figure 4:**
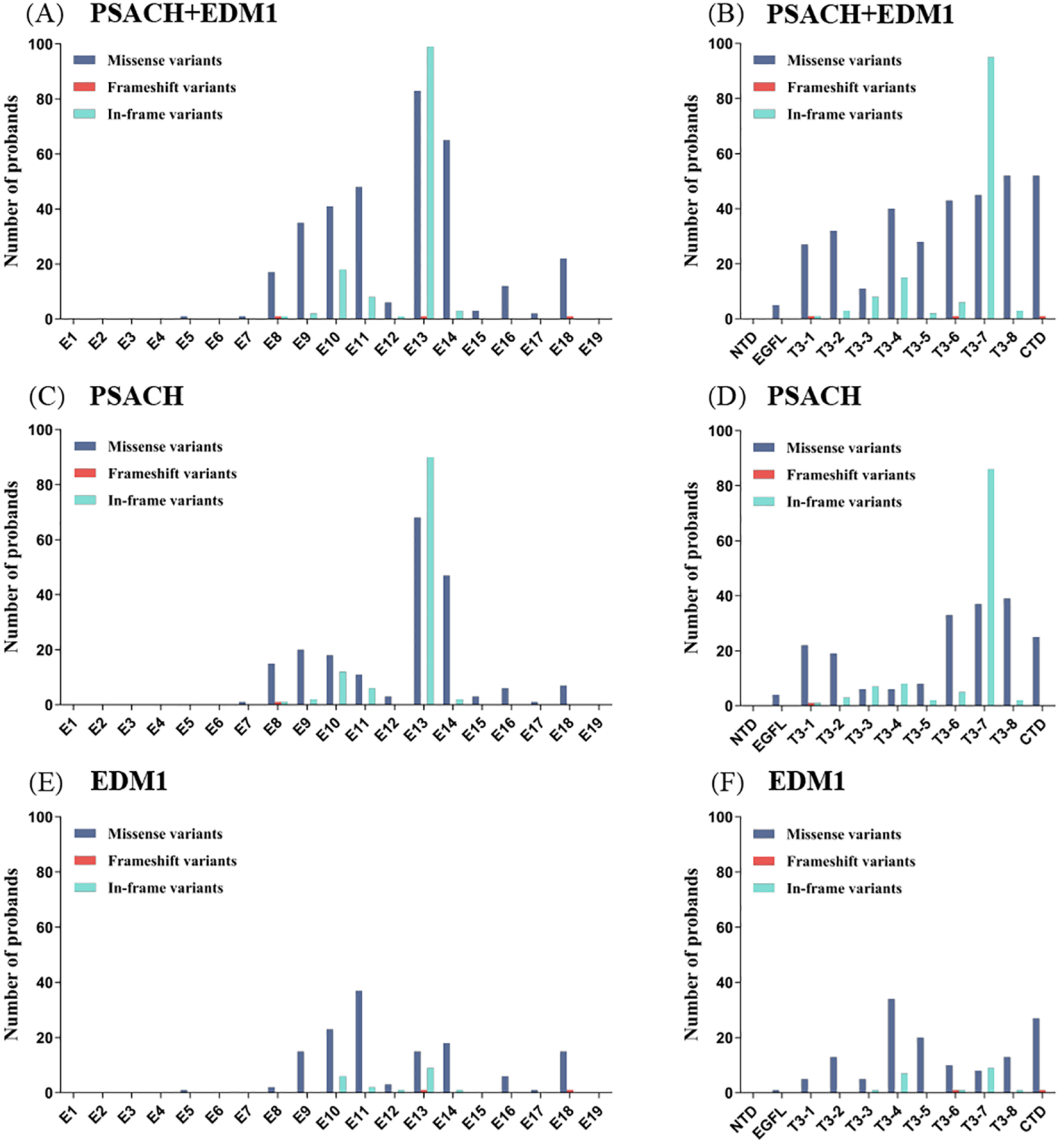
Distribution of variants in 314 PSACH and EDM1 probands. **(A, B)** Distribution of the different types of variants over exons 1–19 of *COMP* gene and domains of COMP protein in 471 PSACH/EDM1 probands. **(C, D)** Distribution of the different types of variants over exons 1–19 of *COMP* gene and domains of COMP protein in 314 PSACH probands. **(E, F)** Distribution of the different types of variants over exons 1–19 of *COMP* gene and domains of COMP protein in 157 EDM1 probands. COMP, cartilage oligomeric matrix protein; PSACH, pseudoachondroplasia; EDM1, multiple epiphyseal dysplasia-1; NTD, N-terminal domain; EGFL, type II epidermal growth factor-like repeats; T3, type III calmodulin-like calcium-binding repeats; CTD, C-terminal domain.

The number and percentage of different variant types in 314 PSACH probands and 157 EDM1 probands were shown in **Figure 3**. Detailed information of variants identified in probands was shown in Supplemental file 2. Although missense variants were the most common variant type in both PSACH and EDM1, the proportion of in-frame variants in PSACH was significantly higher than that in EDM1 (36.3% vs. 12.1%, *p* < 0.001). The distribution of variants over the exons and domains in 471 PSACH/EDM1 probands was illustrated in **Figure 4**. Exon 13 (183 probands, 38.9%) (**Figure 4A**) and type III repeat domain (413 probands, 87.7%) (**Figure 4B**) were the most commonly affected regions, respectively. In addition, the most common hotspot variant was the 3 bp deletion of GAC (c.1417_1419del, p.Asp473del) in exon 13 (T3-7), which caused an in-frame variant in 70 PSACH probands (70/471, 14.9%). Since this hotspot variant only occurred in PSACH probands, the distribution of variants over exons and domains in 314 PSACH probands was similar with that of the whole cohort (**Figures 4C, D**). Nevertheless, both missense variants and in-frame variants in exon 13 and T3 in EDM1 probands were less than that in PSACH probands, leading to the different pattern of variants distribution (**Figures 4E, F**).

### Clinical and radiographic characteristics of patients with PSACH/EDM1

**Figure 5:**
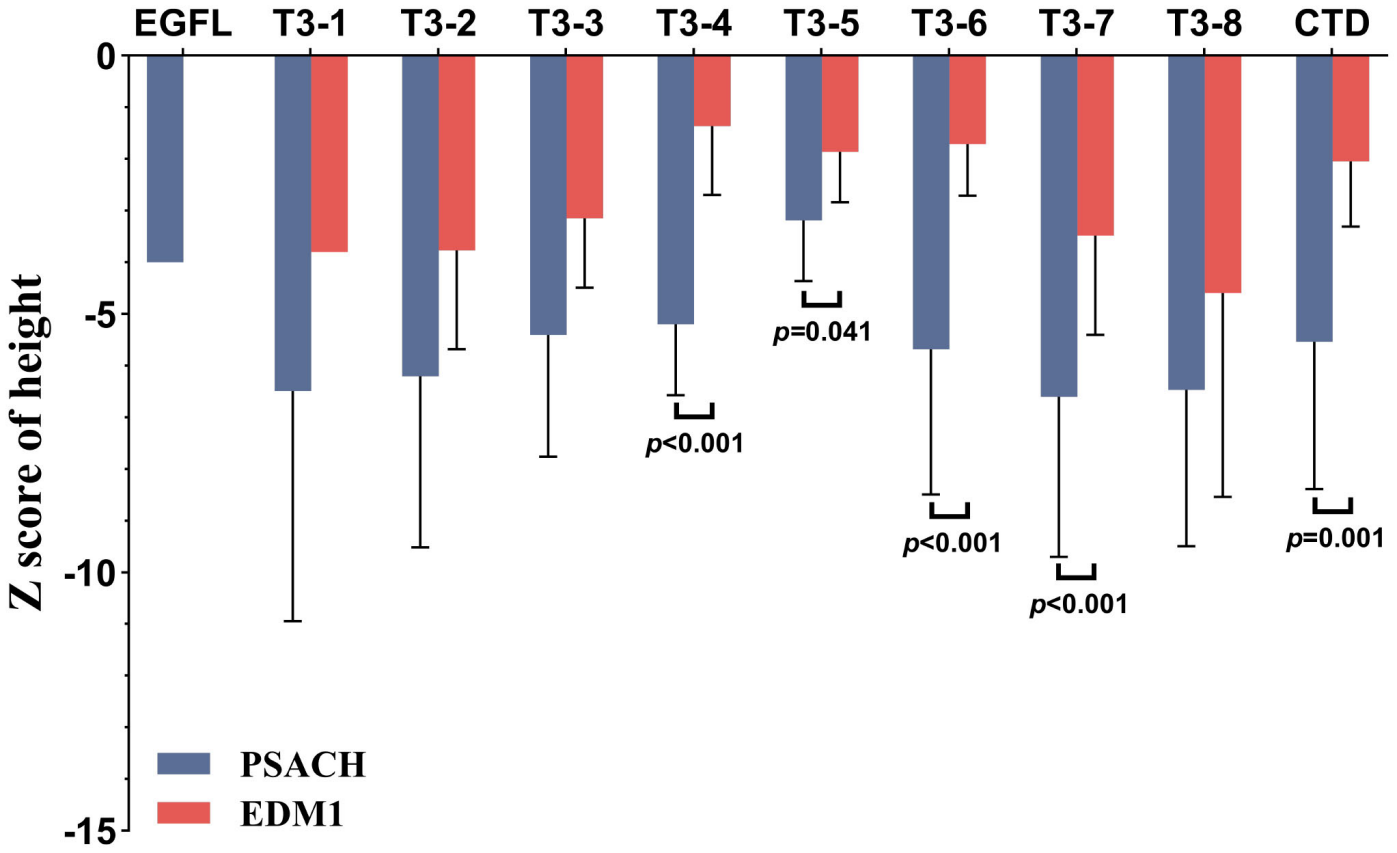
Comparison of Z score of height between patients with PSACH and patients with EDM1 according to the domain of variants. The *p* values were obtained by the independent samples t test for normally distributed data or the Mann-Whitney U test for nonparametric data. Effect sizes (Cohen’s d for parametric data, r for nonparametric data): T3-4 (Cohen’s d = -2.86, 95% CI: -3.97 to -1.72), T3-5 (Cohen’s d = -1.22, 95% CI: -2.36 to -0.05), T3-6 (r = -0.60, 95% CI: -0.76 to -0.37), T3-7 (Cohen’s d = -1.10, 95% CI: -1.57 to -0.63), CTD (Cohen’s d = -1.59, 95% CI: -2.54 to -0.60). Negative values of Cohen’s d and r indicate that Z-score of height in PSACH patients is lower than that in EDM1 patients. PSACH, pseudoachondroplasia; EDM1, multiple epiphyseal dysplasia-1.

The comparison of clinical and radiographic features between patients with PSACH and patients with EDM1 was presented in **Table 1**. Among the patients with relevant information recorded, the median age of the 145 recorded probands with PSACH was younger than that of the 62 recorded probands with EDM1 (8.0 [4.0, 18.5] *vs.* 14.0 [8.6, 20.3] years, *p* < 0.001). In addition, the median onset age of the 75 recorded patients with PSACH was also younger than that of the 42 recorded patients with EDM1 (1.8 [1.0, 2.0] *vs.* 3.0 [1.0, 9.0] years, *p* < 0.001). Male-to-female ratio was not significantly different between two groups (174/157 *vs.* 110/116, *p* = 0.367). Z score of height of the 185 recorded patients with PSACH was significantly lower than that of the 108 recorded patients with EDM1 (-5.5 [-7.9, -3.8] *vs.* -2.1 [-3.2, -1.2], *p* < 0.001). Comparison of Z score of height between patients with PSACH and patients with EDM1 according to the domain of variants was shown in **Figure 5**. The difference of Z score of height between two groups were significant in T3-4 (-5.2 ± 0.6 *vs.* -1.3 ± 0.2, *p* < 0.001), T3-5 (3.2 ± 0.4 *vs.* -1.9 ± 0.4, *p* = 0.041), T3-6 (-4.8 [-7.3, -3.7] *vs.* -1.7 [-2.9, -1.3], *p* < 0.001), T3-7 (-6.6 ± 0.4 vs. -3.5 ± 0.4, *p* < 0.001) and CTD (-5.4 ± 0.9 *vs.* -2.0 ± 0.4, *p* = 0.001). Although there were no statistically significant differences in Z score of height among subgroups within the group, patients with variants in T3–4 and T3–5 tended to exhibit greater height compared to those with variants in other regions in both PSACH and EDM1.

**Table 1:** Comparison of clinical and radiographic features between patients with PSACH and patients with EDM1.

Clinical and radiographic features	Patients with PSACH (n = 474)	Patients with EDM1 (n = 356)	*p* value
General features			
Age of probands at the visit, years	8.0 (4.0, 18.5) (n = 145)	14.0 (8.6, 20.3) (n = 62)	<0.001*
Onset age, years	1.8 (1.0, 2.0) (n = 75)	3.0 (1.0, 9.0) (n = 42)	<0.001*
Gender, male/female	174/157 (n = 331)	110/116 (n = 226)	0.367
Z score of height	-5.5 (-7.9, -3.8) (n = 185)	-2.1 (-3.2, -1.2) (n = 108)	<0.001*
Clinical features	n = 220	n = 146	
Gait abnormality, n (%)	135 (61.4)	69 (47.3)	0.008*
Brachydactyly, n (%)	165 (75.0)	51 (34.9)	<0.001*
Genu varum/valgum, n (%)	136 (60.5)	30 (20.5)	<0.001*
Restricted extension of joints, n (%)	56 (25.5)	26 (17.8)	0.086
Joint laxity, n (%)	57 (25.9)	24 (16.4)	0.041*
Joint pain/osteoarthritis, n (%)	88 (40.0)	102 (69.9)	<0.001*
Pes planus, n (%)	6 (2.7)	24 (16.4)	<0.001*
Radiographic features	n = 187	n =96	
Anterior beaking of the vertebra, n (%)	123 (65.8)	7 (7.3)	<0.001*
Small/irregular/ossification-delayed epiphysis, n (%)	125 (66.8)	58 (60.4)	0.284
Irregular/flared metaphysis, n (%)	157 (84.0)	34 (35.4)	<0.001*
Abnormal femoral head, n (%)	84 (44.9)	59 (61.5)	0.008*
Acetabular dysplasia, n (%)	91 (48.7)	38 (39.6)	0.146
Small/irregular/ossification-delayed carpal bones, n (%)	55 (29.4)	35 (36.5)	0.228

PSACH, pseudoachondroplasia; EDM1, multiple epiphyseal dysplasia-1.

Continuous variables were presented as mean ± SD or median (interquartile range).

Significant values (*p* < 0.05) are presented in bold with *.

Regarding clinical features, we found that the proportion of gait abnormality (61.4% *vs.* 47.3%, *p* = 0.008), brachydactyly (75.0% *vs.* 34.9%, *p* < 0.001), genu varum/valgum (60.5% vs. 20.5%, *p* < 0.001), and joint laxity (25.9% *vs.* 16.4%, *p* = 0.041) in PSACH group was significantly higher than that in EDM1 group. In contrast, the proportion of joint pain/osteoarthritis (40.0% *vs.* 69.9%, *p* < 0.001) and pes planus (2.7% *vs.* 16.4%, *p* < 0.001) in PSACH group was lower than that in EDM1 group.

We also compared the radiographic features between patients with PSACH and patients with EDM1. Both anterior beaking of the vertebra and irregular/flared metaphysis were more common in PSACH group than that in EDM1 group (both *p* < 0.001), while the proportion of small/irregular/ossification-delayed epiphysis was not significantly different between two groups. Although abnormal femoral head is more likely to occur in EDM1 group (*p* = 0.008), both proportions of acetabular dysplasia and small/irregular/ossification-delayed carpal bones were comparable between PSACH group and EDM1 group.

To further support the continuous phenotypic spectrum of PSACH and EDM1, we analyzed the distribution of onset age and Z score of height across patients with complete data on both parameters (**Supplementary Figure 1**). Onset age ranged from 0 to 50 years, with PSACH patients clustering in the earlier range (median 2.0 years) and EDM1 patients clustering in the later range (median 3.0 years), yet with substantial overlap (1.0-2.0 years). Similarly, Z score of height ranged from -15.6 to 0.6, with PSACH patients showing more severe short stature (median -4.6) and EDM1 patients showing milder short stature (median -2.8), without a distinct cutoff between two groups.

### Genotype–phenotype correlation

#### Missense variants in EGF-like domain

Since there was no pathogenic variant found in NTD, we first analyzed the variants in the domain of EGF-like. Up to now, only 3 missense variants (c.500G>A, p.Gly167Glu; c.700C>T, p.Pro234Ser; c.772G>C, p.Gly258Arg) in this domain have been reported in 5 probands with EDM1/PSACH. Only the variant in the domain of EGF-like 2 (c.500G>A, p.Gly167Glu) led to the phenotype of EDM1, while the other two variants in the domain of EGF-like 4 lead to the phenotype of PSACH. Due to the limited number of variants and patients in this domain, more cases need to be further accumulated for analysis of genotype-phenotype correlation.

#### Missense variants in type III repeats domain

**Figure 6:**
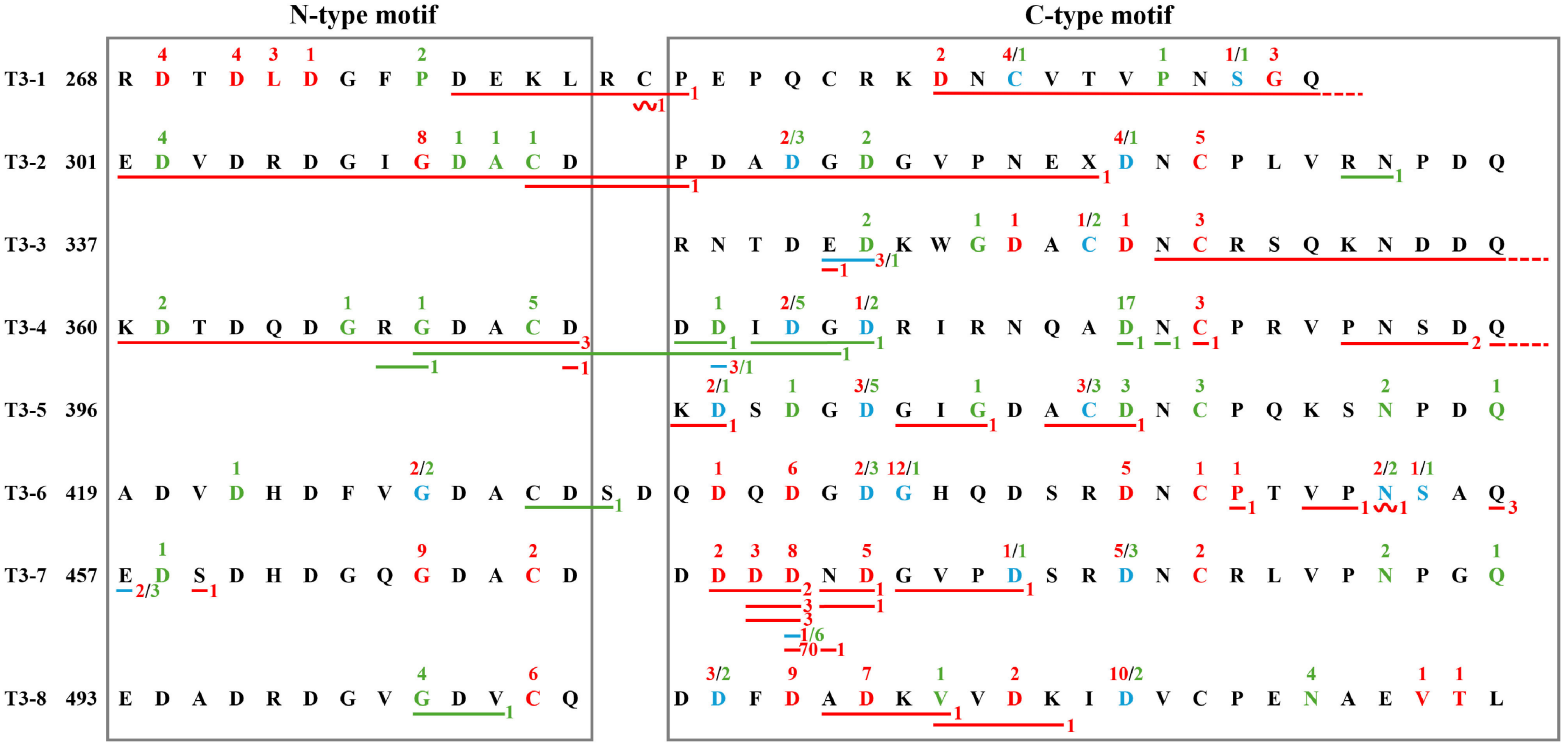
Location and number of missense variants, in-frame variants, and frameshift variants found in 335 probands of PSACH/EDM1 in type III repeat region of COMP. For missense variants, the influenced residues leading to PSACH were marked in red, while those leading to EDM1 were marked in green and those leading to both PSACH and EDM1 were marked in blue. The number of probands with the missense variant is indicated above the relevant residue (red number for PSACH and green number for EDM1). For in-frame variants, the involved residues were underlined using the same color scheme. Dashed underline was used when the in-frame variants cross type III repeat regions. The number of probands with the in-frame variant is indicated after the underline (red number for PSACH and green number for EDM1). Notably, two variants involving Asp472 and Asn473, p.Asp472_Asn473del found in 3 PSACH probands and p.Asp472_Asn473dup found in 3 PSACH probands, were marked separately. Similarly, two variants involving Asp474 and Asn475, p.Asp474_Asn475del found in 1 PSACH probands and p.Asp474_Asn475dup found in 1 PSACH probands, were marked separately. For frameshift variants, the involved residues were wavy underlined using the same color scheme. The number of probands with the frameshift variant is indicated after the wavy underline. COMP, cartilage oligomeric matrix protein; PSACH, pseudoachondroplasia; EDM1, multiple epiphyseal dysplasia-1.

Majority of probands with PSACH/EDM1 (335/471, 71.1%) harbored missense variants. Most of them (278/335, 83.0%) carried missense variants located in type III repeats domain (**Figure 6**), indicating the importance of type III repeats domain for COMP protein. We then analyzed the association between the location of missense variants within the type III repeats domain and the frequency of PSACH versus EDM1 (**Table 2**).

**Table 2:** Association between the location of missense variant within the T3 repeats of COMP and the frequency of PSACH versus EDM1.

Repeat	PSACH		EDM-1		OR (95% CI)	*p* for Chi-square test
	In this repeat	In other repeat	In this repeat	In other repeat		
T3-1	22	148	5	103	3.06 (1.12, 8.35)	0.023*
T3-2	19	151	13	95	0.93 (0.48, 1.80)	0.827
T3-3	6	164	5	103	0.76 (0.24, 2.44)	0.755
T3-4	6	164	34	74	0.11 (0.05, 0.26)	<0.001*
T3-5	8	162	20	88	0.25 (0.12, 0.56)	<0.001*
T3-6	33	137	10	98	2.10 (1.08, 4.08)	0.023*
T3-7	37	133	8	100	2.94 (1.42, 6.07)	0.002*
T3-8	39	131	13	95	1.91 (1.07, 3.40)	0.023*

COMP, cartilage oligomeric matrix protein; PSACH, pseudoachondroplasia; EDM-1, multiple epiphyseal dysplasia-1; OR, odds ratio; CI, confidence interval.

Significant values (*p* < 0.05) are presented in bold with *.

Missense variants in T3–1 showed significant association with a greater frequency of PSACH (22:148 *vs.* 5:103), with an odds ratio (OR) of 3.06 (95% confidence interval [CI]: 1.12-8.35, *p* = 0.023). Several missense variants affecting key residues with high frequency which only found in PSACH contributed to this association, including Asp269 (n = 4), Asp271 (n = 4), Leu272 (n = 3) and Gly299 (n = 3).

Similarly, missense variants in T3-6 (33:137 *vs.* 10:98), T3-7 (37:133 *vs.* 8:100) and T3-8 (39:131 *vs.* 13:95) were significantly associated with a greater frequency of PSACH, with OR values of 2.10 (95% CI: 1.08-4.08, *p* = 0.023), 2.94 (95% CI: 1.42-6.07, *p* = 0.002) and 1.91 (95% CI: 1.07-3.40, *p* = 0.023), respectively. Missense variants affecting the key residues with high frequency which were only found in PSACH contributed to this association, including Asp437 (n = 6), Asp446 (n = 5), Gly465 (n = 9), Asp473 (n = 8), Asp475 (n = 5), Cys504 (n = 6), Asp509 (n = 9), Asp511 (n = 7).

In contrast, missense variants in T3-4 (6:164 *vs.* 34:74) and T3-5 (8:162 *vs.* 20:88) showed a very significant association with EDM1, with OR values of 0.11 (95% CI: 0.05-0.26, *p* < 0.001) and 0.25 (95% CI: 0.12-0.56, *p* < 0.001), respectively. The most frequent missense variant in EDM1, c.1153G>A/T leading the change of Asp385 (n = 17) in T3-4, might explain for this association in T3-4. In addition, there have been no variant in T3–5 reported to occur exclusively in PSACH, which may explain the aforementioned association in T3-5.

Notably, missense variants in T3–2 and T3–3 showed no significant association with either phenotype (T3-2: OR = 0.93, 95% CI: 0.48-1.80, *p* = 0.827; T3-3: OR = 0.76, 95%CI: 0.24-2.44, *p* = 0.755).

#### In-frame variants in type III repeats domain

A total of 38 in-frame variants were summarized in probands with PSACH (n = 114, 85.7%) and probands with EDM1 (n = 19, 14.3%). All the in-frame variants were identified in type III repeats domain (**Figure 6**). The most common in-frame variant was the deletion of an aspartic acid codon (GAC) (c.1417_1419del, p.Asp473del) at nucleotides 1405–1419 encoding five consecutive aspartic acid residues 469–473 of COMP protein. This in-frame variant was identified in 70 probands with PSACH and became the most common variant of *COMP* gene (70/471, 14.9%).

Within the GAC repeat sequence at nucleotides 1405-1419, deletion two (c.1414_1419del, p.Asp472_Asp473del) (n = 3) or three (c.1411_1419del, p.Asp471_Asp473del) (n = 2) aspartic acid residues also led to PSACH. Interestingly, duplication of two aspartic acid residues (c.1414_1419dup, p.Asp472_Asp473dup) only caused PSACH (n = 3), while duplication of one aspartic acid residue (c.1417_1419dup, p.Asp473dup) tended to cause EDM1 (6/7, 85.7%).

In addition to the hotspot variants within the GAC repeat region, two variants also seemed to be PSACH-specific, including c.1048_1116del (p.Asn350_Asp372del) (n = 3) and p.Gln456del (n = 3). Interestingly, there were several recurrent in-frame variants in the T3 region that can lead to either PSACH or EDM1, such as c.1021_1026del (p.Glu341_Asp342del) (n = 3 for PSACH; n = 1 for EDM1) and c.1120_1122del (p.Asp374del) (n = 3 for PSACH; n = 1 for EDM1). Furthermore, an in-frame deletion of three consecutive nucleotides (positions 1369-1373) resulting in the loss of Glu457 was identified in both PSACH (n=2) and EDM1 (n=3) probands.

#### Missense variants in carboxyl-terminal domain

A total of 53 probands (11.3%) with PSACH/EDM1 harbored variants in CTD. With the exception of one frameshift variant (c.2223_2224insC, p.Asn742Glnfs*2) occurred in EDM1, all other variants within the CTD region were missense variants. Several mutation-prone sites have been identified, including Thr529 (n = 9), Asn555 (n = 4), Thr585 (n = 7), Arg718 (n = 16), and Gly719 (n = 5). Pathogenic variants at Gly719 were exclusively observed in PSACH, while alteration of Asn555 were specific to EDM1. Substitution of Thr529 predominantly led to PSACH (*p* < 0.001), whereas alterations of Arg718 were primarily associated with EDM1 (*p* < 0.001).

## Discussion

This study reviewed *COMP* gene variants related to PSACH/EDM1 from 106 previous publications, providing a comprehensive description of the types and locations of *COMP* gene variants in 830 patients, along with clinical information. In addition to English literature, this study incorporated literature published in Chinese (accounting for 15.3% of the included patients), thereby enabling a more comprehensive exploration of genotype-phenotype associations compared to previous study (8).

Data summarized in this review allowed the comparison of clinical data between PSACH and EDM1 with large sample size for the first time, confirming that PSACH manifested earlier age of onset and visit compared to EDM1. Consistent with the statement in previous literature (6), we confirmed that PSACH exhibited more severe clinical manifestations, including shorter stature and higher rates of lower limb deformity, gait abnormality, and joint laxity. In contrast, EDM1 showed a higher rate of joint pain/osteoarthritis. Regarding imaging features, PSACH was characterized by the presence of anterior beaking of the vertebra and a higher rate of metaphyseal involvement compared to EDM1. Additionally, EDM1 exhibited a higher prevalence of abnormal femoral head than PSACH.

COMP plays an important role in stimulating proliferation of chondrocyte, promoting collagen secretion, and enhancing mechanical strength of extracellular matrix (ECM) tissues (9–11). Thus, extensive studies have been conducted to elucidate the pathogenic mechanisms underlying *COMP* gene variants. Prior experimental studies have demonstrated that variants in the T3 repeat compromise calcium binding, and then prevent the protein folding correctly, resulting in endoplasmic reticulum (ER) stress by upregulating CHOP (CCAAT-enhancer-binding protein homologous protein) due to the retention of mutant COMP in the ER of chondrocyte (12, 13). Subsequent studies have further inferred that ER stress then drives oxidative stress and inflammation responses, forming a self-perpetuating stress loop (14). ER stress combined with inflammation is hypothesized to activate mTORC1 signaling, inhibiting autophagy which prevents clearance of mutant COMP (15). In addition, oxidative stress leads to necroptosis of chondrocytes by DNA damage (16). These mechanistic hypotheses suggest that premature loss of chondrocytes, impaired proliferation of chondrocytes and decreased synthesis of ECM in growth plate could be potential contributors to impaired linear growth of bone. Moreover, decreased COMP-mediated collagen secretion into ECM, insufficient ECM replenishment of the articular cartilage, and senescence-driven matrix degradation may be associated with early-onset osteoarthritis. Different from the effect of variants in T3 repeat, the variant in CTD (p.Thr583Met) induced unfolded protein and cell stress response without retention of the mutant COMP in ER, leading to reduced chondrocyte proliferation and increased apoptosis (17). Based on the pathology of PSACH, therapeutic interventions showed potential efficacy in the mouse model of PSACH (MT-COMP mice) including preventing/reducing COMP synthesis, eliminating CHOP expression, reducing inflammation and oxidative stress (18–20). Unfortunately, the only clinical trial exploring the efficacy of resveratrol in PSACH was terminated due to inability to recruit target number (NCT03866200). Thus, there remains a critical unmet clinical need for effective therapies in PSACH/EDM1.

In this review, mutational spectra of *COMP* gene from 471 probands demonstrated that exon 13 exhibited the highest frequency of both missense and in-frame variants. Exon 13 is 182 bp in length, second only to exon 19, which could partially account for the concentration of missense variants in exon 13. The high occurrence rate of in-frame variants in exon 13 was due to the prevalent 3 bp deletion of GAC (c.1417_1419del, p.Asp473del) located in this exon.

The most common type of variant in *COMP* gene in this review was missense variant, mainly located within T3 repeat region. More than half of the probands (278/471, 59.0%) harbored missense variants located in this region, providing the possibility to explore genotype-phenotype associations. In addition to the missense variants in T3-6, T3-7, and T3–8 previously summarized to be more strongly associated with PSACH (8), we found that missense variants in T3–1 were also more likely to cause PSACH due to the accumulation of newly reported PSACH cases with missense variants in T3-1. In contrast, missense variants in T3–4 and T3–5 were more strongly associated with EDM1. This genotype-phenotype statistical correlation indicated that variants in T3–4 and T3–5 may be associated with a less severe phenotype, with the underlying regulatory mechanism, requiring further functional validation. The height trend observed across T3 repeat region aligned with this inference, as patients with variants in T3–4 and T3–5 exhibiting greater height compared to those with variants in other regions in both PSACH and EDM1 shown in **Figure 5**.

**Figure 7:**
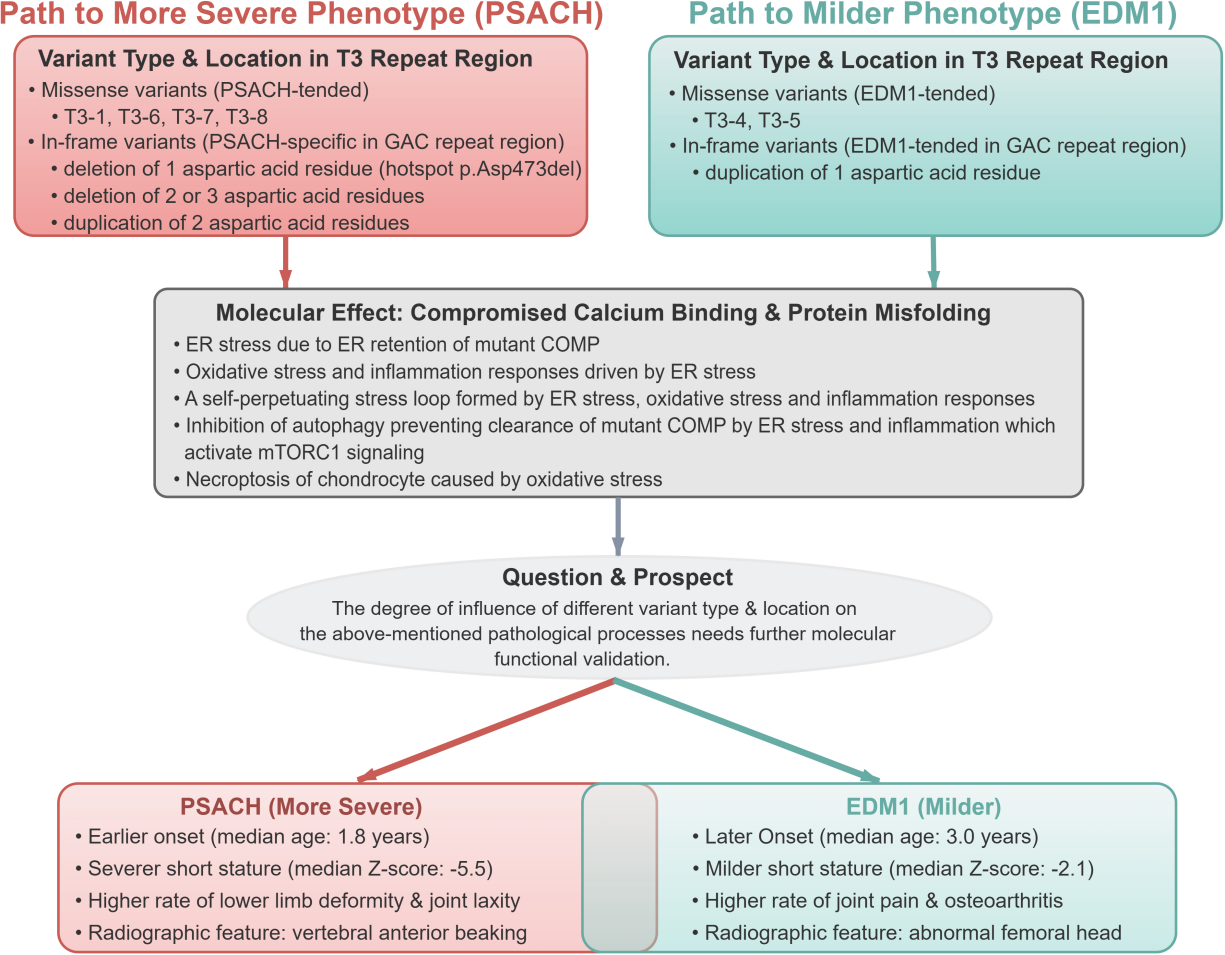
Schematic summary of genotype-pathology-phenotype relationships in COMP-associated dysplasia. COMP, cartilage oligomeric matrix protein; PSACH, pseudoachondroplasia; EDM1, multiple epiphyseal dysplasia-1; T3, type III repeat region.

Second only to missense variants, the next most frequent type of variants was in-frame variant, all of which located in T3 repeat region. Majority of the in-frame variants resulted in PSACH (114/133, 85.7%), indicating that in-frame variants tend to cause greater disruption to the structure of COMP protein even though without frame shift. Among them, deletion of one aspartic acid residue in the GAC repeat region (c.1405-1419) was the most common variant in *COMP* gene which exclusively found in PSACH, along with deletions of two or three aspartic acid residues. In contrast, duplication of one aspartic acid residue was more likely to cause EDM1, while duplication of two aspartic acid residues only caused PSACH. These statistical association findings suggested that the deletion of one aspartic acid residue in the GAC repeat region may have a profounder effect on COMP protein folding than the duplication of one aspartic acid residue, though this inference requires further confirmation through *in vitro* functional experiments or animal models. Furthermore, duplication of two aspartic acid residues may exert a stronger effect on COMP protein folding than duplication of one aspartic acid residue, and this hypothesis remains to be supported by direct molecular functional validation (**Figure 7**).

Frameshift variants, which usually result in premature stop codons, are expected to cause more severe dysfunction of protein than missense variants and in-frame variants. To date, only three frameshift variants of *COMP* gene have been identified in PSACH/EDM1. Interestingly, two of the three frameshift variants were reported in patients with EDM1. A novel frameshift variant in exon 13 (T3-6), c.1359del (p.Asn453Lysfs*62), was recently identified in a EDM1 family (21). The affected family members presented with early-onset osteoarthritis and low-normal height (-1.9SD ~ 0.04SD), even though some of them also had Noonan syndrome caused by variants of *LZTR1* gene. Another frameshift variant, c.2223_2224insC (p.Asn742Glnfs*2) in exon 18 (CTD), was also identified in a 16-year-old girl with EDM1 and her affected mother without detailed clinical information reported (22, 23). The third frameshift variant is c.846_867 + 3del (p.Cys282Trpfs*5) in exon 8 (T3-1) reported in 6-year-old girl with PSACH presented with short stature and windswept deformity of the lower limbs, whose unaffected mother presented low-level somatic mosaicism (5%) (24). Notably, the PSACH-associated frameshift variant in exon 8 yields a severely truncated protein, whereas the EDM1-linked frameshift variants in exons 13 and 20 produce proteins that are only mildly truncated and retain most T3 repeats. This structural distinction explains their phenotypic divergence. The fact that these truncations cause EDM1, a stark contrast to the multitude of PSACH-causing missense variants, collectively suggests that the key factor dictating PSACH versus EDM1 is not variant type per se, but rather the variant’s propensity to cause core domain misfolding, secretory incapacity, and the resultant gain-of-toxicity (dominant-negative effect) within the chondrocytes.

Both PSACH and EDM1 were inherited in an autosomal dominant manner, as heterozygous variants in the *COMP* gene are well known causes. To date, only one study reported a biallelic COMP variant (c.1423G>A, p.Asp475Asn) in two individuals with severe PSACH in a four-generation consanguineous family, while heterozygosity for the variant in other family members (25). The phenotypes of heterozygous and homozygous individuals presented in this study comply with a gene dose dependent phenotype, and an additive effect of two mutant alleles.

A critical insight from this review is that PSACH and EDM1 constitute a continuous phenotypic spectrum rather than strictly dichotomous diseases. This spectrum is supported by two lines of evidence: first, the overlapping clinical features, such as lower limb deformity and short stature, are present in both conditions but differ in frequency and severity; second, the shared causative variants, such as c.1021_1026del, can manifest as either PSACH or EDM1. Mechanistically, this spectrum is likely driven by the degree of COMP protein dysfunction induced by different variants: variants that severely impair calcium binding, protein folding, and extracellular secretion tend to cluster at the PSACH end of the spectrum, while variants with milder effects on protein function are more commonly associated with EDM1. The phenotypic heterogeneity among patients with same variants suggests that factors beyond the variant itself, such as genetic modifiers, environmental factors, or stochastic events, may modulate the severity of the phenotype, reinforcing the concept that PSACH and EDM1 are not dichotomous but interconnected entities.

There are some limitations in this study. First, this study is a comprehensive review focused on case aggregation rather than a strict systematic review, thus formal risk of bias assessment with standard tools was not performed. This is primarily due to the heterogeneous nature of included literature (predominantly case reports/case series with incomplete clinical data and inconsistent reporting standards) and the study’s core goal of summarizing variant distribution and preliminary genotype-phenotype correlations. To mitigate potential biases, we implemented strict quality control (e.g., Sanger/NGS-verified *COMP* variants, exclusion of ambiguous/duplicate data, stratified data usage), but residual biases (e.g., publication bias favoring more severe phenotypes) may still exist and should be considered when interpreting results. Second, some cases diagnosed based solely on clinical manifestation and radiographic findings were excluded, which may miss a part of cases. Thirdly, the differences of severity between EDM1 and PSACH should be interpreted with caution due to the predominantly cross-sectional nature of the included studies and the age-related longitudinal progression of osteoarthritis and functional disability in EDM1. Fourthly, future studies should focus on identifying genetic modifiers or environmental factors that influence the position of individuals along the PSACH-EDM1 spectrum, as well as exploring the functional gradient of *COMP* variants to better explain the continuum of phenotypic severity. Finally, only published articles in English and Chinese in PubMed, CNKI, and Wanfang were searched, so the analysis might not be comprehensive.

In conclusion, PSACH and EDM1 represent a continuous phenotypic spectrum rather than strictly dichotomous entities, characterized by distinct but overlapping clinical and imaging features, that pose challenges for differential diagnosis. This review enrolling 830 patients confirmed that PSACH exhibits more severe phenotypic manifestations compared with EDM1. Genotype-phenotype correlation analysis revealed that in-frame variants in T3 repeat region are more strongly associated with PSACH, with the most common variant (p.Asp473del) being PSACH specific. Missense variants in T3–1 and T3–6 to T3–8 showed stronger associations with PSACH, whereas those in T3–4 and T3–5 were more related with EDM1. Recognizing the continuous spectrum of PSACH and EDM1, combined with the genotype-phenotype correlations identified in this study, will assist clinicians in recognizing the clinical and imaging characteristics of these rare conditions and facilitating accurate diagnosis.
