# Supplementary material for: Genotype-phenotype correlations in PSACH/EDM1 patients with COMP gene variants: a comprehensive review of 830 cases
Source: Front Endocrinol (Lausanne). 2026 Feb 19;17:1740770. doi: 10.3389/fendo.2026.1740770 (PMC12960193; doi:10.3389/fendo.2026.1740770)
Supplement: Supplementary file 1 [file Table1.docx]

**Supplemental file 1** Literatures published in English identifying the clinical and genetic information of patients with PSACH/EDM1

[1] Mazzotti A, Artioli E, Brizola E*, et al*. Multiple Osteochondritis Dissecans as Main Manifestation of Multiple Epiphyseal Dysplasia Caused by a Novel Cartilage Oligomeric Matrix Protein Pathogenic Variant: A Clinical Report. *Genes (Basel)*. 2024;15(11):1490.

[2] Sun H, Zhang G, Li N, Bu X. Molecular diagnosis of patients with syndromic short stature identified by trio whole-exome sequencing. *Front Genet*. 2024;15:1399186.

[3] Zhou L, Chen J, Liu Q, Yang S, Xie W, Peng Y. Case Report: Whole-exome sequencing identified two novel COMP variants causing pseudoachondroplasia. *Front Endocrinol (Lausanne)*. 2023;14:1267946.

[4] Hasegawa K, Futagawa N, Ago Y*, et al*. Novel and recurrent COMP gene variants in five Japanese patients with pseudoachondroplasia: skeletal changes from the neonatal to infantile periods. *Clin Pediatr Endocrinol*. 2023;32(4):221-227.

[5] Yin L, Zhu Y, Jiang W*, et al*. Functional characterization of a rare pathogenic variant c.875G > A, p.(Cys292Tyr) in COMP. *Ann Hum Genet*. 2023;87(5):241-247.

[6] Toral López J, González Huerta LM. Recurrent Mutation (p.Arg718Pro) in the COMP Gene with Clinical Heterogeneity of Pseudoachondroplasia. *Mol Syndromol*. 2023;14(4):341-346.

[7] Zhao Q, Li Y, Shao Q*, et al*. Clinical and genetic evaluation of children with short stature of unknown origin. *BMC Med Genomics*. 2023;16(1):194.

[8] Pálla S, Anker P, Farkas K*, et al*. Co-occurrence of neurofibromatosis type 1 and pseudoachondroplasia - a first case report. *BMC Pediatr*. 2023;23(1):110.

[9] Al Kaissi A, Nassib N, Bouchoucha S*, et al*. Torticollis in Connection with Spine Phenotype. *Diagnostics (Basel)*. 2022;12(7):1672.

[10] Jacob P, Bhavani GSL, Shah H*, et al*. Pseudoachondroplasia: Phenotype and genotype in 11 Indian patients. *Am J Med Genet A*. 2022;188(3):751-759.

[11] Liang H, Hou Y, Pang Q*, et al*. Clinical, Biochemical, Radiological, Genetic and Therapeutic Analysis of Patients with COMP Gene Variants. *Calcif Tissue Int*. 2022;110(3):313-323.

[12] Chen M, Miao H, Liang H*, et al*. Clinical Characteristics of Short-Stature Patients With Collagen Gene Mutation and the Therapeutic Response to rhGH. *Front Endocrinol (Lausanne)*. 2022;13:820001.

[13] Qiu J, Tan J. In vitro and in silico analysis of a E559K mutation on cartilage oligomeric matrix protein. *Mutat Res*. 2022;824:111774.

[14] Hsu RH, Chien YH, Hwu WL, Lee NC. Diversity in heritable disorders of connective tissue at a single center. *Connect Tissue Res*. 2021;62(5):580-585.

[15] Tuncel G, Akcan N, Gul S*, et al*. Identification of a Novel De Novo COMP Gene Variant as a Likely Cause of Pseudoachondroplasia. *Appl Immunohistochem Mol Morphol*. 2021;29(7):546-550.

[16] Kim SJ, Lee SM, Choi JM*, et al*. Genetic Analysis Using a Next Generation Sequencing-Based Gene Panel in Patients With Skeletal Dysplasia: A Single-Center Experience. *Front Genet*. 2021;12:670608.

[17] Guo BB, Jin JY, Yuan ZZ, Zeng L, Xiang R. A Novel COMP Mutated Allele Identified in a Chinese Family with Pseudoachondroplasia. *Biomed Res Int*. 2021;2021:6678531.

[18] El-Lababidi N, Zikánová M, Baxová A*, et al*. Age Dependent Progression of Multiple Epiphyseal Dysplasia and Pseudoachondroplasia Due to Heterozygous Mutations in COMP Gene. *Prague Med Rep*. 2020;121(3):153-162.

[19] Shao J, Zhao S, Yan Z*, et al*. A novel COMP mutation in a Chinese family with multiple epiphyseal dysplasia. *BMC Med Genet*. 2020;21(1):115.

[20] Ansari H, Mohammadi-Asl J, Hajjari M*, et al*. Exome sequencing revealed a p.G299R mutation in the COMP gene in an Iranian family suffering from pseudoachondroplasia. *J Gene Med*. 2019;21(8):e3103.

[21] Yang L, Zhang C, Wang W*, et al*. Pathogenic gene screening in 91 Chinese patients with short stature of unknown etiology with a targeted next-generation sequencing panel. *BMC Med Genet*. 2018;19(1):212.

[22] Lin WD, Chou IC, Wang CH, Tsai FJ. Novel mutations in the cartilage oligomeric matrix protein gene identified in two Taiwanese patients with pseudoachondroplasia and multiple epiphyseal dysplasia. *Pediatr Neonatol*. 2018;59(4):412-414.

[23] Ichihashi Y, Takagi M, Ishii T, Watanabe K, Nishimura G, Hasegawa T. Two novel mutations of COMP in Japanese boys with pseudoachondroplasia. *Hum Genome Var*. 2018;5:12.

[24] Tamaro G, Pederiva F, Dibello D*, et al*. A Child with Diminished Linear Growth and Waddling Gait. *J Pediatr*. 2018;201:297-297.e291.

[25] Chen J, Zhang W, He J, Zhang R, Cao Y, Liu X. A novel mutation in exon 11 of COMP gene in a Chinese family with pseudoachondroplasia. *Genes Dis*. 2019;6(1):47-55.

[26] Tariq M, Khan TN, Lundin L*, et al*. Homozygosity for a missense variant in COMP gene associated with severe pseudoachondroplasia. *Clin Genet*. 2018;93(1):182-186.

[27] Gu C, Yang Z, Tan H, Zhang Y, Lu Y, Ma Y. Decreased Plasma COMP and Increased Plasma CTX-II Levels in a Chinese Pseudoachondroplasia Family with Novel COMP Mutation. *Biomed Res Int*. 2017;2017:5809787.

[28] Sakamoto Y, Yamamoto T, Kajino Y*, et al*. Multiple epiphyseal dysplasia mimicking osteoarthritis due to acetabular dysplasia: A report of a familial case with a COMP mutation. *J Orthop Sci*. 2017;22(5):967-971.

[29] Ideta H, Uchiyama S, Hayashi M, Kosho T, Nakamura Y, Kato H. Painful locking of the wrist in a patient with pseudoachondroplasia confirmed by COMP mutation. *J Surg Case Rep*. 2017;2017(1):rjw216.

[30] Yu WJ, Zhang Z, He JW, Fu WZ, Wang C, Zhang ZL. Identification of two novel mutations in the COMP gene in six families with pseudoachondroplasia. *Mol Med Rep*. 2016;14(3):2180-2186.

[31] Luo H, Yu S, Lin Y*, et al*. A novel deleterious mutation in the COMP gene that causes pseudoachondroplasia. *Hum Genome Var*. 2016;3:16009.

[32] Zhang H, Yang R, Wang Y*, et al*. A pilot study of gene testing of genetic bone dysplasia using targeted next-generation sequencing. *J Hum Genet*. 2015;60(12):769-776.

[33] Briggs MD, Brock J, Ramsden SC, Bell PA. Genotype to phenotype correlations in cartilage oligomeric matrix protein associated chondrodysplasias. *Eur J Hum Genet*. 2014;22(11):1278-1282.

[34] Donkervoort S, Schindler A, Tesi-Rocha C*, et al*. 'Double trouble': diagnostic challenges in Duchenne muscular dystrophy in patients with an additional hereditary skeletal dysplasia. *Neuromuscul Disord*. 2013;23(12):955-961.

[35] Singh A, Abiramalatha T, Pradhan G, Jin DK, Kapoor S. A report of an Indian boy with a delayed diagnosis of pseudochondroplasia. *J Clin Diagn Res*. 2013;7(7):1479-1481.

[36] Xie X, Liao L, Gao J, Luo X. A novel COMP mutation in a Chinese patient with pseudoachondroplasia. *Gene*. 2013;522(1):102-106.

[37] Liu FX, Li YX, Zhang XD, Ren CA, Huang SZ, Yu MX. EDM1: a novel point mutation in cartilage oligomeric matrix protein gene in a Chinese family with multiple epiphyseal dysplasia. *Chin Med J (Engl)*. 2013;126(6):1103-1107.

[38] Jackson GC, Mittaz-Crettol L, Taylor JA*, et al*. Pseudoachondroplasia and multiple epiphyseal dysplasia: a 7-year comprehensive analysis of the known disease genes identify novel and recurrent mutations and provides an accurate assessment of their relative contribution. *Hum Mutat*. 2012;33(1):144-157.

[39] Kim OH, Park H, Seong MW*, et al*. Revisit of multiple epiphyseal dysplasia: ethnic difference in genotypes and comparison of radiographic features linked to the COMP and MATN3 genes. *Am J Med Genet A*. 2011;155a(11):2669-2680.

[40] Cao LH, Wang LB, Wang SS, Ma HW, Ji CY, Luo Y. Identification of novel and recurrent mutations in the calcium binding type III repeats of cartilage oligomeric matrix protein in patients with pseudoachondroplasia. *Genet Mol Res*. 2011;10(2):955-963.

[41] Dai L, Xie L, Wang Y*, et al*. A novel COMP mutation in a pseudoachondroplasia family of Chinese origin. *BMC Med Genet*. 2011;12:72.

[42] Jung WW, Balce GC, Cho JW, Jung SC, Hong SJ, Song HR. COMP and Col9A3 mutations and their relationship to the pseudoachondroplasia phenotype. *Int J Mol Med*. 2010;26(6):885-891.

[43] Elliott AM, Bocangel P, Reed MH, Greenberg CR. A novel COMP mutation in an Inuit patient with pseudoachondroplasia and severe short stature. *Genet Mol Res*. 2010;9(3):1785-1790.

[44] Liu FX, Li ZL, Wei ZJ*, et al*. Genetic analysis and serum level of cartilage oligomeric matrix protein in patients with pseudoachondroplasia. *Chin Med J (Engl)*. 2010;123(16):2181-2184.

[45] Wang CH, Lin WD, Tsai A, Tsai FJ. Novel human pathological mutations. Gene symbol: COMP. Disease: pseudoachondroplasia. *Hum Genet*. 2009;125(3):350.

[46] Vatanavicharn N, Lachman RS, Rimoin DL. Multilayered patella: similar radiographic findings in pseudoachondroplasia and recessive multiple epiphyseal dysplasia. *Am J Med Genet A*. 2008;146a(13):1682-1686.

[47] Tufan AC, Satiroglu-Tufan NL, Jackson GC, Semerci CN, Solak S, Yagci B. Serum or plasma cartilage oligomeric matrix protein concentration as a diagnostic marker in pseudoachondroplasia: differential diagnosis of a family. *Eur J Hum Genet*. 2007;15(10):1023-1028.

[48] Kennedy J, Jackson GC, Barker FS*, et al*. Novel and recurrent mutations in the C-terminal domain of COMP cluster in two distinct regions and result in a spectrum of phenotypes within the pseudoachondroplasia -- multiple epiphyseal dysplasia disease group. *Hum Mutat*. 2005;25(6):593-594.

[49] Kennedy J, Jackson G, Ramsden S*, et al*. COMP mutation screening as an aid for the clinical diagnosis and counselling of patients with a suspected diagnosis of pseudoachondroplasia or multiple epiphyseal dysplasia. *Eur J Hum Genet*. 2005;13(5):547-555.

[50] Jakkula E, Mäkitie O, Czarny-Ratajczak M*, et al*. Mutations in the known genes are not the major cause of MED; distinctive phenotypic entities among patients with no identified mutations. *Eur J Hum Genet*. 2005;13(3):292-301.

[51] Nakashima E, Mabuchi A, Kubota M*, et al*. Novel and recurrent exon 13 mutations of COMP in pseudoachondroplasia. *Am J Med Genet A*. 2005;132a(1):108-109.

[52] Song HR, Li QW, Oh CW, Lee KS, Koo SK, Jung SC. Mesomelic dwarfism in pseudoachondroplasia. *J Pediatr Orthop B*. 2004;13(5):340-344.

[53] Hecht JT, Makitie O, Hayes E*, et al*. Chondrocyte cell death and intracellular distribution of COMP and type IX collagen in the pseudoachondroplasia growth plate. *J Orthop Res*. 2004;22(4):759-767.

[54] Jakkula E, Lohiniva J, Capone A*, et al*. A recurrent R718W mutation in COMP results in multiple epiphyseal dysplasia with mild myopathy: clinical and pathogenetic overlap with collagen IX mutations. *J Med Genet*. 2003;40(12):942-948.

[55] Nakayama H, Endo Y, Aota S, Sato M, Fujita T, Kikuchi S. Novel mutations of the cartilage oligomeric matrix protein (COMP) gene in two Japanese patients with pseudoachondroplasia. *Oncol Rep*. 2003;10(4):871-873.

[56] Mabuchi A, Manabe N, Haga N*, et al*. Novel types of COMP mutations and genotype-phenotype association in pseudoachondroplasia and multiple epiphyseal dysplasia. *Hum Genet*. 2003;112(1):84-90.

[57] Song HR, Lee KS, Li QW, Koo SK, Jung SC. Identification of cartilage oligomeric matrix protein (COMP) gene mutations in patients with pseudoachondroplasia and multiple epiphyseal dysplasia. *J Hum Genet*. 2003;48(5):222-225.

[58] Shotelersuk V, Punyashthiti R. A novel mutation of the COMP gene in a Thai family with pseudoachondroplasia. *Int J Mol Med*. 2002;9(1):81-84.

[59] Unger S, Korkko J, Krakow D, Lachman RS, Rimoin DL, Cohn DH. Double heterozygosity for pseudoachondroplasia and spondyloepiphyseal dysplasia congenita. *Am J Med Genet*. 2001;104(2):140-146.

[60] Mabuchi A, Haga N, Ikeda T*, et al*. Novel mutation in exon 18 of the cartilage oligomeric matrix protein gene causes a severe pseudoachondroplasia. *Am J Med Genet*. 2001;104(2):135-139.

[61] Vranka J, Mokashi A, Keene DR*, et al*. Selective intracellular retention of extracellular matrix proteins and chaperones associated with pseudoachondroplasia. *Matrix Biol*. 2001;20(7):439-450.

[62] Newman B, Donnah D, Briggs MD. Molecular diagnosis is important to confirm suspected pseudoachondroplasia. *J Med Genet*. 2000;37(1):64-65.

[63] Deere M, Sanford T, Francomano CA, Daniels K, Hecht JT. Identification of nine novel mutations in cartilage oligomeric matrix protein in patients with pseudoachondroplasia and multiple epiphyseal dysplasia. *Am J Med Genet*. 1999;85(5):486-490.

[64] Délot E, King LM, Briggs MD, Wilcox WR, Cohn DH. Trinucleotide expansion mutations in the cartilage oligomeric matrix protein (COMP) gene. *Hum Mol Genet*. 1999;8(1):123-128.

[65] Loughlin J, Irven C, Mustafa Z*, et al*. Identification of five novel mutations in cartilage oligomeric matrix protein gene in pseudoachondroplasia and multiple epiphyseal dysplasia. *Hum Mutat*. 1998;Suppl 1:S10-17.

[66] Ikegawa S, Ohashi H, Nishimura G*, et al*. Novel and recurrent COMP (cartilage oligomeric matrix protein) mutations in pseudoachondroplasia and multiple epiphyseal dysplasia. *Hum Genet*. 1998;103(6):633-638.

[67] Deere M, Sanford T, Ferguson HL, Daniels K, Hecht JT. Identification of twelve mutations in cartilage oligomeric matrix protein (COMP) in patients with pseudoachondroplasia. *Am J Med Genet*. 1998;80(5):510-513.

[68] Délot E, Brodie SG, King LM, Wilcox WR, Cohn DH. Physiological and pathological secretion of cartilage oligomeric matrix protein by cells in culture. *J Biol Chem*. 1998;273(41):26692-26697.

[69] Hecht JT, Deere M, Putnam E*, et al*. Characterization of cartilage oligomeric matrix protein (COMP) in human normal and pseudoachondroplasia musculoskeletal tissues. *Matrix Biol*. 1998;17(4):269-278.

[70] Briggs MD, Mortier GR, Cole WG*, et al*. Diverse mutations in the gene for cartilage oligomeric matrix protein in the pseudoachondroplasia-multiple epiphyseal dysplasia disease spectrum. *Am J Hum Genet*. 1998;62(2):311-319.

[71] Susic S, Ahier J, Cole WG. Pseudoachondroplasia due to the substitution of the highly conserved Asp482 by Gly in the seventh calmodulin-like repeat of cartilage oligomeric matrix protein. *Hum Mutat*. 1998;Suppl 1:S125-127.

[72] Maddox BK, Keene DR, Sakai LY*, et al*. The fate of cartilage oligomeric matrix protein is determined by the cell type in the case of a novel mutation in pseudoachondroplasia. *J Biol Chem*. 1997;272(49):30993-30997.

[73] Susic S, McGrory J, Ahier J, Cole WG. Multiple epiphyseal dysplasia and pseudoachondroplasia due to novel mutations in the calmodulin-like repeats of cartilage oligomeric matrix protein. *Clin Genet*. 1997;51(4):219-224.

[74] Ballo R, Briggs MD, Cohn DH, Knowlton RG, Beighton PH, Ramesar RS. Multiple epiphyseal dysplasia, ribbing type: a novel point mutation in the COMP gene in a South African family. *Am J Med Genet*. 1997;68(4):396-400.

[75] Ferguson HL, Deere M, Evans R, Rotta J, Hall JG, Hecht JT. Mosaicism in pseudoachondroplasia. *Am J Med Genet*. 1997;70(3):287-291.

[76] Cohn DH, Briggs MD, King LM*, et al*. Mutations in the cartilage oligomeric matrix protein (COMP) gene in pseudoachondroplasia and multiple epiphyseal dysplasia. *Ann N Y Acad Sci*. 1996;785:188-194.

[77] Briggs MD, Hoffman SM, King LM*, et al*. Pseudoachondroplasia and multiple epiphyseal dysplasia due to mutations in the cartilage oligomeric matrix protein gene. *Nat Genet*. 1995;10(3):330-336.

[78] Hecht JT, Nelson LD, Crowder E*, et al*. Mutations in exon 17B of cartilage oligomeric matrix protein (COMP) cause pseudoachondroplasia. *Nat Genet*. 1995;10(3):325-329.
