# Supplementary material for: Genotype-phenotype correlations in PSACH/EDM1 patients with COMP gene variants: a comprehensive review of 830 cases
Source: Front Endocrinol (Lausanne). 2026 Feb 19;17:1740770. doi: 10.3389/fendo.2026.1740770 (PMC12960193; doi:10.3389/fendo.2026.1740770)
Supplement: Supplementary file 2 [file Table2.docx]

**Supplemental file 2** Variants of *COMP* gene identified in PSACH/EDM1 probands

| **Reference** | **Language** | **Disease** | **Exon** | **Change of nucleotide** | **Change of amino acid** | **Domain** |
| --- | --- | --- | --- | --- | --- | --- |
| Jackson, et al. Hum Mutat, 2012 | English | MED | 5 | c.500G>A | p.Gly167Glu | EGF-like 2 |
| Jackson, et al. Hum Mutat, 2012 | English | PSACH | 7 | c.700C>T | p.Pro234Ser | EGF-like 4 |
| Yu, et al. Mol Med Rep, 2016 | English | PSACH | 8 | c.772G>C | p.Gly258Arg | EGF-like 4 |
| Briggs, et al. Eur J Hum Genet, 2014 | English | PSACH | 8 | c.772G>C | p.Gly258Arg | EGF-like 4 |
| Lv, et al. Chin J of Endocrinol Metab, 2019 | Chinese | PSACH | 8 | c.772G>C | p.Gly258Arg | EGF-like 4 |
| Liang et al, Calcif Tissue Int, 2022 | English | PSACH | 8 | c.805G>A | p.Asp269Asn | T3-1 |
| Ideta, et al. J Surg Case Rep, 2017 | English | PSACH | 8 | c.806A>G | p.Asp269Gly | T3-1 |
| Briggs, et al. Eur J Hum Genet, 2014 | English | PSACH | 8 | c.806A>G | p.Asp269Gly | T3-1 |
| Briggs, et al. Eur J Hum Genet, 2014 | English | PSACH | 8 | c.806A>G | p.Asp269Gly | T3-1 |
| Deere, et al. Am J Med Genet, 1999 | English | PSACH | 8 | c.811G>C | p.Asp271His | T3-1 |
| Yu, et al. Mol Med Rep, 2016 | English | PSACH | 8 | c.812A>T | p.Asp271Val | T3-1 |
| Elliott, et al. Genet Mol Res, 2010 | English | PSACH | 8 | c.812A>T | p.Asp271Val | T3-1 |
| Lv, et al. Chin J of Endocrinol Metab, 2019 | Chinese | PSACH | 8 | c.812A>T | p.Asp271Val | T3-1 |
| Deere, et al. Am J Med Genet, 1999 | English | PSACH | 8 | c.815T>C | p.Leu272Pro | T3-1 |
| Liu, et al. Peking Union Medical College, 2010 | Chinese | PSACH | 8 | c.815T>C | p.Leu272Pro | T3-1 |
| Xia, et al. Chin Med J (Engl), 2010 | English | PSACH | 8 | c.815T>C | p.Leu272Pro | T3-1 |
| Briggs, et al. Eur J Hum Genet, 2014 | English | PSACH | 8 | c.818A>C | p.Asp273Ala | T3-1 |
| Jackson, et al. Hum Mutat, 2012 | English | MED | 8 | c.827C>G | p.Pro276Arg | T3-1 |
| Kennedy, et al. Eur J Hum Genet, 2005 | English | MED | 8 | c.827C>G | p.Pro276Arg | T3-1 |
| Kennedy, et al. Eur J Hum Genet, 2005 | English | PSACH | 8 | c.831_851del | p.Asp277_Pro283del | T3-1 |
| Tamaro, et al. J Pediatr, 2018 | English | PSACH | 8 | c.846_867+3del | p.Cys282Trpfs*5 | T3-1 |
| Ikegawa, et al. Hum Genet, 1998 | English | PSACH | 9 | c.868G>A | p.Asp290Asn | T3-1 |
| Jackson, et al. Hum Mutat, 2012 | English | PSACH | 9 | c.869A>G | p.Asp290Gly | T3-1 |
| Jacob et al. Am J Med Genet A, 2022 | English | PSACH | 9 | c.874T>C | p.Cys292Arg | T3-1 |
| Liang et al, Calcif Tissue Int, 2022 | English | PSACH | 9 | c.874T>C | p.Cys292Arg | T3-1 |
| Kim, et al. Am J Med Genet A, 2011 | English | MED | 9 | c.874T>G | p.Cys292Gly | T3-1 |
| Yin, et al. Ann Hum Genet, 2023 | English | PSACH | 9 | c.875G>A | p.Cys292Tyr | T3-1 |
| Deere, et al. Am J Med Genet, 1999 | English | PSACH | 9 | c.876C>G | p.Cys292Trp | T3-1 |
| Briggs, et al. Eur J Hum Genet, 2014 | English | MED | 9 | c.886C>A | p.Pro296Thr | T3-1 |
| Jackson, et al. Hum Mutat, 2012 | English | MED | 9 | c.893C>T | p.Ser298Leu | T3-1 |
| Kennedy, et al. Eur J Hum Genet, 2005 | English | PSACH | 9 | c.893C>T | p.Ser298Leu | T3-1 |
| Ikegawa, et al. Hum Genet, 1998 | English | PSACH | 9 | c.895G>A | p.Gly299Arg | T3-1 |
| Jackson, et al. Hum Mutat, 2012 | English | PSACH | 9 | c.895G>C | p.Gly299Arg | T3-1 |
| Ansari, et al. J Gene Med, 2019 | English | PSACH | 9 | c.896G>A | p.Gly299Arg | T3-1 |
| Kim, et al. Am J Med Genet A, 2011 | English | MED | 9 | c.904G>T | p.Asn302Tyr | T3-2 |
| Briggs, et al. Eur J Hum Genet, 2014 | English | MED | 9 | c.905A>T | p.Asp302Val | T3-2 |
| Deere, et al. Am J Med Genet, 1999 | English | MED | 9 | c.905A>T | p.Asp302Val | T3-2 |
| Deere, et al. Am J Med Genet, 1999 | English | MED | 9 | c.905A>T | p.Asp302Val | T3-2 |
| Mabuchi, et al. Hum Genet, 2003 | English | PSACH | 9 | c.917_IVS9–58del | Del exon 9 | T3-2 |
| Jacob et al. Am J Med Genet A, 2022 | English | PSACH | 9 | c.925G>A | p.Gly309Arg | T3-2 |
| Liang et al, Calcif Tissue Int, 2022 | English | PSACH | 9 | c.925G>A | p.Gly309Arg | T3-2 |
| Chen, et al. Front Endocrinol, 2022 | English | PSACH | 9 | c.925G>A | p.Gly309Arg | T3-2 |
| Wang, et al. Int J Orthop, 2014 | Chinese | PSACH | 9 | c.925G>A | p.Gly309Arg | T3-2 |
| Kennedy, et al. Eur J Hum Genet, 2005 | English | PSACH | 9 | c.925G>A | p.Gly309Arg | T3-2 |
| Délot, et al. J Biol Chem, 1998 | English | PSACH | 9 | c.925G>A | p.Gly309Arg | T3-2 |
| Nakayama, et al. Oncol Rep, 2003 | English | PSACH | 9 | c.925G>C | p.Gly309Arg | T3-2 |
| Kennedy, et al. Eur J Hum Genet, 2005 | English | MED | 9 | c.929A>T | p.Asp310Val | T3-2 |
| Jackson, et al. Hum Mutat, 2012 | English | MED | 9 | c.932C>A | p.Ala311Asp | T3-2 |
| Ichihashi, et al. Hum Genome Var, 2018 | English | PSACH | 9 | c.934_942del | p.Cys312_Pro314del | T3-2 |
| Briggs, et al. Eur J Hum Genet, 2014 | English | MED | 9 | c.935G>A | p.Cys312Tyr | T3-2 |
| Kim, et al. Am J Med Genet A, 2011 | English | MED | 9 | c.949G>A | p.Asn317Asn | T3-2 |
| Zhao, et al. BMC Med Genomics, 2023 | English | MED | 9 | c.949G>T | p.Asp317Tyr | T3-2 |
| Xu, et al. Journal of Chongqing Medical University, 2022 | Chinese | PSACH | 9 | c.950A>C | p.Asp317Ala | T3-2 |
| Kaissi, et al. Diagnostics, 2022 | English | PSACH | 9 | c.950A>G | p.Asp317Gly | T3-2 |
| Jackson, et al. Hum Mutat, 2012 | English | MED | 9 | c.950A>G | p.Asp317Gly | T3-2 |
| Kim, et al. Am J Med Genet A, 2011 | English | MED | 9 | c.955G>C | p.Asn319His | T3-2 |
| Wang, et al. Chin J Med Genet, 2013 | Chinese | MED | 9 | c.956A>T | p.Asp319Val | T3-2 |
| Jacob et al. Am J Med Genet A, 2022 | English | PSACH | 10 | c.976G>A | p.Asp326Asn | T3-2 |
| Yu, et al. Mol Med Rep, 2016 | English | PSACH | 10 | c.976G>A | p.Asp326Asn | T3-2 |
| Lv, et al. Chin J of Endocrinol Metab, 2019 | Chinese | PSACH | 10 | c.976G>A | p.Asp326Asn | T3-2 |
| Jackson, et al. Hum Mutat, 2012 | English | PSACH | 10 | c.976G>T | p.Asp326Tyr | T3-2 |
| Jackson, et al. Hum Mutat, 2012 | English | MED | 10 | c.977A>G | p.Asp326Gly | T3-2 |
| Briggs, et al. Nat Genet, 1995 | English | PSACH | 10 | c.982T>C | p.Cys328Arg | T3-2 |
| Song, et al. J Pediatr Orthop B, 2004 | English | PSACH | 10 | c.982T>C | p.Cys328Arg | T3-2 |
| Cohn, et al. Ann N Y Acad Sci, 1996 | English | PSACH | 10 | c.982T>C | p.Cys328Arg | T3-2 |
| Jung, et al. Int J Mol Med, 2010 | English | PSACH | 10 | c.982T>C | p.Cys328Arg | T3-2 |
| Xu, et al. Journal of Chongqing Medical University, 2022 | Chinese | PSACH | 10 | c.984C>G | p.Cys328Trp | T3-2 |
| Zhong, et al. J Chin Pract Diagn Ther, 2019 | Chinese | PSACH | 10 | c.995_999delinsTG | p.Arg332_Asn333delinsL | T3-2 |
| Jung, et al. Int J Mol Med, 2010 | English | PSACH | 10 | c.1021_1023del | p.Gly341del | T3-3 |
| Kim, et al. Am J Med Genet A, 2011 | English | MED | 10 | c.1021_1026del | p.Glu341_Asp342del | T3-3 |
| Jackson, et al. Hum Mutat, 2012 | English | PSACH | 10 | c.1021_1026del | p.Glu341_Asp342del | T3-3 |
| Kennedy, et al. Eur J Hum Genet, 2005 | English | PSACH | 10 | c.1021_1026del | p.Glu341_Asp342del | T3-3 |
| Kennedy, et al. Eur J Hum Genet, 2005 | English | PSACH | 10 | c.1021_1026del | p.Glu341_Asp342del | T3-3 |
| Cohn, et al. Ann N Y Acad Sci, 1996 | English | MED | 10 | c.1024G>T | p.Asp342Tyr | T3-3 |
| Briggs, et al. Nat Genet, 1995 | English | MED | 10 | c.1024G>T | p.Asp342Tyr | T3-3 |
| Briggs, et al. Eur J Hum Genet, 2014 | English | MED | 10 | c.1034G>A | p.Gly345Asp | T3-3 |
| Maddox, et al. J Biol Chem, 1997 | English | PSACH | 10 | c.1036G>A | p.Asp346Asn | T3-3 |
| Unger, et al. Am J Med Genet, 2001 | English | PSACH | 10 | c.1042T>C | p.Cys348Arg | T3-3 |
| Jackson, et al. Hum Mutat, 2012 | English | MED | 10 | c.1043G>T | p.Cys348Phe | T3-3 |
| Briggs, et al. Eur J Hum Genet, 2014 | English | MED | 10 | c.1044C>G | p.Cys348Trp | T3-3 |
| Ikegawa, et al. Hum Genet, 1998 | English | PSACH | 10 | c.1046A>G | p.Asp349Val | T3-3 |
| Zhang, et al. Chinese Journal of Birth Health & Heredity, 2022 | Chinese | PSACH | 10 | c.1048_1116del | p.Asn350_Asp372del | T3-3 |
| Lu, et al. Chin J Contemp Pediatr, 2013 | Chinese | PSACH | 10 | c.1048_1116del | p.Asn350_Asp372del | T3-3 |
| Jackson, et al. Hum Mutat, 2012 | English | PSACH | 10 | c.1048_1116del | p.Asn350_Asp372del | T3-3 |
| Mabuchi, et al. Hum Genet, 2003 | English | PSACH | 10 | c.1052G>A | p.Cys351Tyr | T3-3 |
| Hasegawa, et al. Clin Pediatr Endocrinol, 2023 | English | PSACH | 10 | c.1052G>A | p.Cys351Tyr | T3-3 |
| Kennedy, et al. Eur J Hum Genet, 2005 | English | PSACH | 10 | c.1052G>A | p.Cys351Tyr | T3-3 |
| Loughlin, et al. Hum Mutat, 1998 | English | MED | 10 | c.1081G>T | p.Asp361Tyr | T3-4 |
| Ikegawa, et al. Hum Genet, 1998 | English | MED | 10 | c.1082A>T | p.Asp361Val | T3-4 |
| Kennedy, et al. Eur J Hum Genet, 2005 | English | MED | 10 | c.1096G>C | p.Gly366Arg | T3-4 |
| Loughlin, et al. Hum Mutat, 1998 | English | MED | 10 | c.1099_1104del | p.Arg367_Gly368del | T3-4 |
| Wang, et al. Sun Yat-sen University, 2009 | Chinese | MED | 10 | c.1102_1131del | p.Gly368_Gly377del | T3-4 |
| Kennedy, et al. Eur J Hum Genet, 2005 | English | MED | 10 | c.1102G>C | p.Gly368Arg | T3-4 |
| Susic, et al. Clin Genet, 1997 | English | MED | 10 | c.1111T>A | p.Cys371Ser | T3-4 |
| Jackson, et al. Hum Mutat, 2012 | English | MED | 10 | c.1111T>A | p.Cys371Ser | T3-4 |
| Kim, et al. Am J Med Genet A, 2011 | English | MED | 10 | c.1112G>A | p.Cys371Tyr | T3-4 |
| Jackson, et al. Hum Mutat, 2012 | English | MED | 10 | c.1112G>A | p.Cys371Tyr | T3-4 |
| Mabuchi, et al. Hum Genet, 2003 | English | MED | 10 | c.1112G>T | p.Cys371Phe | T3-4 |
| Kennedy, et al. Eur J Hum Genet, 2005 | English | PSACH | 10 | c.1114_1116del | p.Asp372del | T3-4 |
| Kim, et al. Am J Med Genet A, 2011 | English | MED | 10 | c.1117_1122del | p.Asp373_Asp374del | T3-4 |
| Jackson, et al. Hum Mutat, 2012 | English | MED | 10 | c.1120G>A | p.Asp374Asn | T3-4 |
| Kim, et al. Am J Med Genet A, 2011 | English | MED | 10 | c.1120_1122del | p.Asp374del | T3-4 |
| Briggs, et al. Am J Hum Genet, 1998 | English | PSACH | 10 | c.1120_1122del | p.Asp374del | T3-4 |
| Cohn, et al. Ann N Y Acad Sci, 1996 | English | PSACH | 10 | c.1120_1122del | p.Asp374del | T3-4 |
| Briggs, et al. Nat Genet, 1995 | English | PSACH | 10 | c.1120_1122del | p.Asp374del | T3-4 |
| Liang et al, Calcif Tissue Int, 2022 | English | MED | 10 | c.1123_1134del | p.Ile375_Asp378del | T3-4 |
| Briggs, et al. Eur J Hum Genet, 2014 | English | MED | 10 | c.1126G>A | p.Asp376Asn | T3-4 |
| Hou, et al. Journal of Sun Yat-sen University (Medical Sciences), 2024 | Chinese | MED | 10 | c.1126G>A | p.Asp376Asn | T3-4 |
| Jackson, et al. Hum Mutat, 2012 | English | MED | 10 | c.1126G>A | p.Asp376Asn | T3-4 |
| Kim, et al. Am J Med Genet A, 2011 | English | MED | 10 | c.1126G>C | p.Asp376His | T3-4 |
| El-Lababidi, et al. Prague Med Rep, 2020 | English | PSACH | 10 | c.1126G>T | p.Asp376Tyr | T3-4 |
| Wang, et al. Chin J Med Genet, 2019 | Chinese | MED | 10 | c.1126G>T | p.Asp376Tyr | T3-4 |
| Kennedy, et al. Eur J Hum Genet, 2005 | English | PSACH | 10 | c.1127A>T | p.Asp376Val | T3-4 |
| Kennedy, et al. Eur J Hum Genet, 2005 | English | MED | 10 | c.1132G>T | p.Asp378Tyr | T3-4 |
| Briggs, et al. Eur J Hum Genet, 2014 | English | MED | 10 | c.1133A>T | p.Asp378Val | T3-4 |
| Jackson, et al. Hum Mutat, 2012 | English | PSACH | 10 | c.1133A>T | p.Asp378Val | T3-4 |
| Jackson, et al. Hum Mutat, 2012 | English | MED | 11 | c.1152_1154del | p.Asp385del | T3-4 |
| Song, et al. J Hum Genet, 2003 | English | MED | 11 | c.1153G>A | p.Asp385Asn | T3-4 |
| Kim, et al. Am J Med Genet A, 2011 | English | MED | 11 | c.1153G>A | p.Asp385Asn | T3-4 |
| Kim, et al. Am J Med Genet A, 2011 | English | MED | 11 | c.1153G>A | p.Asp385Asn | T3-4 |
| Kim, et al. Am J Med Genet A, 2011 | English | MED | 11 | c.1153G>A | p.Asp385Asn | T3-4 |
| Kim, et al. Am J Med Genet A, 2011 | English | MED | 11 | c.1153G>A | p.Asp385Asn | T3-4 |
| Mabuchi, et al. Hum Genet, 2003 | English | MED | 11 | c.1153G>A | p.Asp385Asn | T3-4 |
| Lou, et al. Chin J Orthop, 2020 | Chinese | MED | 11 | c.1153G>A | p.Asp385Asn | T3-4 |
| Jackson, et al. Hum Mutat, 2012 | English | MED | 11 | c.1153G>A | p.Asp385Asn | T3-4 |
| Jackson, et al. Hum Mutat, 2012 | English | MED | 11 | c.1153G>A | p.Asp385Asn | T3-4 |
| Jackson, et al. Hum Mutat, 2012 | English | MED | 11 | c.1153G>A | p.Asp385Asn | T3-4 |
| Kennedy, et al. Eur J Hum Genet, 2005 | English | MED | 11 | c.1153G>A | p.Asp385Asn | T3-4 |
| Kennedy, et al. Eur J Hum Genet, 2005 | English | MED | 11 | c.1153G>A | p.Asp385Asn | T3-4 |
| Kennedy, et al. Eur J Hum Genet, 2005 | English | MED | 11 | c.1153G>A | p.Asp385Asn | T3-4 |
| Kennedy, et al. Eur J Hum Genet, 2005 | English | MED | 11 | c.1153G>A | p.Asp385Asn | T3-4 |
| Shao, et al. BMC Med Genet, 2020 | English | MED | 11 | c.1153G>T | p.Asp385Tyr | T3-4 |
| Shao, et al. Peking Union Medical College, 2022 | Chinese | MED | 11 | c.1153G>T | p.Asp385Tyr | T3-4 |
| Jackson, et al. Hum Mutat, 2012 | English | MED | 11 | c.1153G>T | p.Asp385Tyr | T3-4 |
| Kennedy, et al. Eur J Hum Genet, 2005 | English | MED | 11 | c.1154_1156del | p.Asn386del | T3-4 |
| Duan, et al. Chin J Appl Clin Pediatr, 2020 | Chinese | PSACH | 11 | c.1159T>C | p.Cys387Arg | T3-4 |
| Jackson, et al. Hum Mutat, 2012 | English | PSACH | 11 | c.1159T>C | p.Cys387Arg | T3-4 |
| Ikegawa, et al. Hum Genet, 1998 | English | PSACH | 11 | c.1159T>G | p.Cys387Gly | T3-4 |
| Luo, et al. Hum Genome Var, 2016 | English | PSACH | 11 | c.1159_1161del | p.Cys387del | T3-4 |
| Kennedy, et al. Eur J Hum Genet, 2005 | English | PSACH | 11 | c.1170_1181delinsTGT | p.Pro391_Asp394delinsV | T3-4 |
| Loughlin, et al. Hum Mutat, 1998 | English | PSACH | 11 | c.1170_1181delinsTGT | p.Pro391_Asp394delinsV | T3-4 |
| Deere, et al. Am J Med Genet, 1999 | English | PSACH | 11 | c.1183_1191del | p.Gln395_Asp397del | T3-4至T3-5 |
| Jackson, et al. Hum Mutat, 2012 | English | MED | 11 | c.1189G>C | p.Asp397His | T3-5 |
| Cao, et al. Genet Mol Res, 2011 | English | PSACH | 11 | c.1189G>T | p.Asp397Tyr | T3-5 |
| Li, et al. Chin J Contemp Pediatr, 2013 | Chinese | PSACH | 11 | c.1189G>T | p.Asp397Tyr | T3-5 |
| Kennedy, et al. Eur J Hum Genet, 2005 | English | MED | 11 | c.1195G>A | p.Asp399Asn | T3-5 |
| Yang, et al. BMC Med Genet, 2018 | English | MED | 11 | c.1201G>A | p.Asp401Asn | T3-5 |
| Wang, et al. Shanxi University, 2018 | Chinese | MED | 11 | c.1201G>A | p.Asp401Asn | T3-5 |
| Yang, et al. Chinese Journal of Practical Pediatrics, 2017 | Chinese | MED | 11 | c.1201G>A | p.Asp401Asn | T3-5 |
| Ni, et al. Nanjing Normal University, 2017 | Chinese | PSACH | 11 | c.1201G>A | p.Asp401Asn | T3-5 |
| Kennedy, et al. Eur J Hum Genet, 2005 | English | MED | 11 | c.1201G>A | p.Asp401Asn | T3-5 |
| Jacob et al. Am J Med Genet A, 2022 | English | PSACH | 11 | c.1201G>C | p.Asp401His | T3-5 |
| Jacob et al. Am J Med Genet A, 2022 | English | PSACH | 11 | c.1201G>T | p.Asp401Tyr | T3-5 |
| Briggs, et al. Eur J Hum Genet, 2014 | English | MED | 11 | c.1201G>T | p.Asp401Tyr | T3-5 |
| Jackson, et al. Hum Mutat, 2012 | English | PSACH | 11 | c.1205_1212delinsTCTGT | p.Gly402_Gly404delinsVC | T3-5 |
| Jackson, et al. Hum Mutat, 2012 | English | MED | 11 | c.1210G>A | p.Gly404Arg | T3-5 |
| Lv, et al. Chin J of Endocrinol Metab, 2019 | Chinese | PSACH | 11 | c.1215_1223del | p.Ala406_Asp408del | T3-5 |
| Chen, et al. Genes Dis, 2018 | English | PSACH | 11 | c.1219T>C | p.Cys407Arg | T3-5 |
| El-Lababidi, et al. Prague Med Rep, 2020 | English | MED | 11 | c.1220G>A | p.Cys407Tyr | T3-5 |
| El-Lababidi, et al. Prague Med Rep, 2020 | English | MED | 11 | c.1220G>A | p.Cys407Tyr | T3-5 |
| Cao, et al. Genet Mol Res, 2011 | English | PSACH | 11 | c.1220G>A | p.Cys407Tyr | T3-5 |
| Li, et al. Chin J Contemp Pediatr, 2013 | Chinese | PSACH | 11 | c.1220G>A | p.Cys407Tyr | T3-5 |
| Kennedy, et al. Eur J Hum Genet, 2005 | English | MED | 11 | c.1220G>T | p.Cys407Phe | T3-5 |
| Briggs, et al. Eur J Hum Genet, 2014 | English | MED | 11 | c.1222G>A | p.Asp408Asn | T3-5 |
| Liang et al, Calcif Tissue Int, 2022 | English | MED | 11 | c.1222G>T | p.Asp408Tyr | T3-5 |
| Loughlin, et al. Hum Mutat, 1998 | English | MED | 11 | c.1222G>T | p.Asp408Tyr | T3-5 |
| Kim, et al. Am J Med Genet A, 2011 | English | MED | 11 | c.1229G>A | p.Cus410Tyr | T3-5 |
| Jackson, et al. Hum Mutat, 2012 | English | MED | 11 | c.1229G>A | p.Cys410Tyr | T3-5 |
| Jackson, et al. Hum Mutat, 2012 | English | MED | 11 | c.1229G>A | p.Cys410Tyr | T3-5 |
| Jackson, et al. Hum Mutat, 2012 | English | MED | 11 | c.1245C>G | p.Asn415Lys | T3-5 |
| Jackson, et al. Hum Mutat, 2012 | English | MED | 11 | c.1245C>G | p.Asn415Lys | T3-5 |
| Kim, et al. Am J Med Genet A, 2011 | English | MED | 11 | c.1252C>G | p.Gln418Glu | T3-5 |
| Jakkula, et al. Eur J Hum Genet, 2005 | English | MED | 12 | c.1265A>C | p.Asp422Ala | T3-6 |
| Deere, et al. Am J Med Genet, 1998 | English | PSACH | 12 | c.1280G>A | p.Gly427Glu | T3-6 |
| Délot, et al. J Biol Chem, 1998 | English | PSACH | 12 | c.1280G>A | p.Gly427Glu | T3-6 |
| Jackson, et al. Hum Mutat, 2012 | English | MED | 12 | c.1280G>A | p.Gly427Glu | T3-6 |
| Briggs, et al. Eur J Hum Genet, 2014 | English | MED | 12 | c.1280G>C | p.Gly427Ala | T3-6 |
| Jackson, et al. Hum Mutat, 2012 | English | MED | 12 | c.1289_1294delinsTGTGGT | p.Cys430_Ser432delinsLWC | T3-6 |
| Zhou, et al.Front Endocrinol, 2023 | English | PSACH | 12 | c.1304A>T | p.Asp435Val | T3-6 |
| Jacob et al. Am J Med Genet A, 2022 | English | PSACH | 13 | c.1309G>A | p.Asp437Asn | T3-6 |
| Hasegawa, et al. Clin Pediatr Endocrinol, 2023 | English | PSACH | 13 | c.1309G>T | p.Asp437Tyr | T3-6 |
| Briggs, et al. Eur J Hum Genet, 2014 | English | PSACH | 13 | c.1310A>G | p.Asp437Gly | T3-6 |
| Mabuchi, et al. Hum Genet, 2003 | English | PSACH | 13 | c.1310A>G | p.Asp437Gly | T3-6 |
| Deere, et al. Am J Med Genet, 1999 | English | PSACH | 13 | c.1310A>G | p.Asp437Gly | T3-6 |
| Xu, et al. Journal of Chongqing Medical University, 2022 | Chinese | PSACH | 13 | c.1310A>T | p.Asp437Val | T3-6 |
| Kennedy, et al. Eur J Hum Genet, 2005 | English | MED | 13 | c.1315G>A | p.Asp439Asn | T3-6 |
| Zhang, et al. J Hum Genet, 2015 | English | MED | 13 | c.1316A>G | p.Asn439Gly | T3-6 |
| Briggs, et al. Eur J Hum Genet, 2014 | English | MED | 13 | c.1317C>A | p.Asp439Glu | T3-6 |
| Liang et al, Calcif Tissue Int, 2022 | English | PSACH | 13 | c.1317C>G | p.Asp439Glu | T3-6 |
| Guo, et al. Biomed Res Int, 2021 | English | PSACH | 13 | c.1317C>G | p.Asp439Glu | T3-6 |
| Kim, et al. Am J Med Genet A, 2011 | English | MED | 13 | c.1318G>A | p.Gly440Arg | T3-6 |
| Jackson, et al. Hum Mutat, 2012 | English | PSACH | 13 | c.1318G>A | p.Gly440Arg | T3-6 |
| Kennedy, et al. Eur J Hum Genet, 2005 | English | PSACH | 13 | c.1318G>A | p.Gly440Arg | T3-6 |
| Kennedy, et al. Eur J Hum Genet, 2005 | English | PSACH | 13 | c.1318G>A | p.Gly440Arg | T3-6 |
| Loughlin, et al. Hum Mutat, 1998 | English | PSACH | 13 | c.1318G>A | p.Gly440Arg | T3-6 |
| Briggs, et al. Am J Hum Genet, 1998 | English | PSACH | 13 | c.1318G>A | p.Gly440Arg | T3-6 |
| Cao, et al. Genet Mol Res, 2011 | English | PSACH | 13 | c.1318G>C | p.Gly440Arg | T3-6 |
| Briggs, et al. Am J Hum Genet, 1998 | English | PSACH | 13 | c.1319G>A | p.Gly440Glu | T3-6 |
| Pálla, et al. BMC Pediatr, 2023 | English | PSACH | 13 | c.1319G>A | p.Gly440Glu | T3-6 |
| Briggs, et al. Am J Hum Genet, 1998 | English | PSACH | 13 | c.1319G>A | p.Gly440Glu | T3-6 |
| Cohn, et al. Ann N Y Acad Sci, 1996 | English | PSACH | 13 | c.1319G>A | p.Gly440Glu | T3-6 |
| Cohn, et al. Ann N Y Acad Sci, 1996 | English | PSACH | 13 | c.1319G>A | p.Gly440Glu | T3-6 |
| Zhou, et al.Front Endocrinol, 2023 | English | PSACH | 13 | c.1319G>T | p.Gly440Val | T3-6 |
| El-Lababidi, et al. Prague Med Rep, 2020 | English | PSACH | 13 | c.1336G>A | p.Asp446Asn | T3-6 |
| Jackson, et al. Hum Mutat, 2012 | English | PSACH | 13 | c.1336G>A | p.Asp446Asn | T3-6 |
| Briggs, et al. Eur J Hum Genet, 2014 | English | PSACH | 13 | c.1336G>C | p.Asp446His | T3-6 |
| Xu, et al. Journal of Chongqing Medical University, 2022 | Chinese | PSACH | 13 | c.1337A>G | p.Asp446Gly | T3-6 |
| Hasegawa, et al. Clin Pediatr Endocrinol, 2023 | English | PSACH | 13 | c.1337A>G | p.Asp446Gly | T3-6 |
| Jackson, et al. Hum Mutat, 2012 | English | PSACH | 13 | c.1343G>C | p.Cys448Ser | T3-6 |
| Deere, et al. Am J Med Genet, 1998 | English | PSACH | 13 | c.1345C>A | p.Pro449Thr | T3-6 |
| Shotelersuk, et al. Int J Mol Med, 2002 | English | PSACH | 13 | c.1345-1347del | p.Pro449del | T3-6 |
| Dai, et al. BMC Med Genet, 2011 | English | PSACH | 13 | c.1352_1353insTGTCCCTGG | p.451Val_452ProinsVPG | T3-6 |
| Cohn, et al. Ann N Y Acad Sci, 1996 | English | MED | 13 | c.1358A>G | p.Asn453Ser | T3-6 |
| Briggs, et al. Am J Hum Genet, 1998 | English | MED | 13 | c.1358A>G | p.Asn453Ser | T3-6 |
| El-Lababidi, et al. Prague Med Rep, 2020 | English | PSACH | 13 | c.1359C>A | p.Asn453Lys | T3-6 |
| Briggs, et al. Eur J Hum Genet, 2014 | English | PSACH | 13 | c.1359C>A | p.Asn453Lys | T3-6 |
| Sun, et al. Front Genet, 2024 | English | MED | 13 | c.1359del | p.Asn453Lysfs*62 | T3-6 |
| Nakashima, et al. Am J Med Genet A, 2005 | English | PSACH | 13 | c.1360A>C | p.Ser454Arg | T3-6 |
| Kennedy, et al. Eur J Hum Genet, 2005 | English | MED | 13 | c.1362T>G | p.Ser454Arg | T3-6 |
| Jung, et al. Int J Mol Med, 2010 | English | PSACH | 13 | c.1366_1368del | p.Gln456del | T3-6 |
| Jung, et al. Int J Mol Med, 2010 | English | PSACH | 13 | c.1366_1368del | p.Gly456del | T3-6 |
| Jung, et al. Int J Mol Med, 2010 | English | PSACH | 13 | c.1366_1368del | p.Gly456del | T3-6 |
| Newman, et al. J Med Genet, 2000 | English | PSACH | 13 | c.1369_1371del | p.Glu457del | T3-7 |
| Ferguson, et al. Am J Med Genet, 1997 | English | PSACH | 13 | c.1370_1372del | p.Glu457del | T3-7 |
| Kim, et al. Am J Med Genet A, 2011 | English | MED | 13 | c.1371_1373del | p.Glu457del | T3-7 |
| Mabuchi, et al. Hum Genet, 2003 | English | MED | 13 | c.1371_1373del | p.Glu457del | T3-7 |
| Jackson, et al. Hum Mutat, 2012 | English | MED | 13 | c.1371_1373del | p.Glu457del | T3-7 |
| Kim, et al. Am J Med Genet A, 2011 | English | MED | 13 | c.1372G>T | p.Asp458Tyr | T3-7 |
| Hecht, et al. Nat Genet, 1995 | English | PSACH | 13 | c.1375_1377del | p.Ser459del | T3-7 |
| Chen, et al. Front Endocrinol, 2022 | English | PSACH | 13 | c.1393G>A | p.Gly465Ser | T3-7 |
| Briggs, et al. Eur J Hum Genet, 2014 | English | PSACH | 13 | c.1393G>A | p.Gly465Ser | T3-7 |
| Xu, et al. Journal of Chongqing Medical University, 2022 | Chinese | PSACH | 13 | c.1393G>A | p.Gly465Ser | T3-7 |
| Kennedy, et al. Eur J Hum Genet, 2005 | English | PSACH | 13 | c.1393G>C | p.Gly465Arg | T3-7 |
| Newman, et al. J Med Genet, 2000 | English | PSACH | 13 | c.1393G>T | p.Gly465Cys | T3-7 |
| Briggs, et al. Eur J Hum Genet, 2014 | English | PSACH | 13 | c.1394G>A | p.Gly465Asp | T3-7 |
| Lin, et al. Pediatr Neonatol, 2018 | English | PSACH | 13 | c.1394G>T | p.Gly465Val | T3-7 |
| Li, et al. Chin J Clinicians, 2024 | Chinese | PSACH | 13 | c.1394G>T | p.Gly465Val | T3-7 |
| Wang, et al. Hum Genet, 2009 | English | PSACH | 13 | c.1394G>T | p.Gly465Val | T3-7 |
| Hecht, et al. Nat Genet, 1995 | English | PSACH | 13 | c.1403G>A | p.Cys468Tyr | T3-7 |
| Xu, et al. Journal of Chongqing Medical University, 2022 | Chinese | PSACH | 13 | c.1403G>A | p.Cys468Thr | T3-7 |
| Xia, et al. Chin Med J (Engl), 2010 | English | PSACH | 13 | c.1411_1419del | p.Asp471_Asp473del | T3-7 |
| Zhang, et al. Peking Union Medical College, 2010 | Chinese | PSACH | 13 | c.1411_1419del | p.Asp471_Asp473del | T3-7 |
| Nakashima, et al. Am J Med Genet A, 2005 | English | PSACH | 13 | c.1412A>C | p.Asp471Ala | T3-7 |
| Kennedy, et al. Eur J Hum Genet, 2005 | English | PSACH | 13 | c.1412A>G | p.Asp471Gly | T3-7 |
| Jung, et al. Int J Mol Med, 2010 | English | PSACH | 13 | c.1414G>T | p.Asp472Tyr | T3-7 |
| Hecht, et al. Nat Genet, 1995 | English | PSACH | 13 | c.1414G>T | p.Asp472Tyr | T3-7 |
| Briggs, et al. Eur J Hum Genet, 2014 | English | PSACH | 13 | c.1414G>C | p.Asp472His | T3-7 |
| Briggs, et al. Eur J Hum Genet, 2014 | English | PSACH | 13 | c.1414_1419del | p.Asp472_Asp473del | T3-7 |
| Jackson, et al. Hum Mutat, 2012 | English | PSACH | 13 | c.1414_1419del | p.Asp472_Asp473del | T3-7 |
| Song, et al. J Pediatr Orthop B, 2004 | English | PSACH | 13 | c.1414_1419del | p.Asp472_Asp473del | T3-7 |
| Deere, et al. Am J Med Genet, 1999 | English | PSACH | 13 | c.1414_1419dup | p.Asp472_Asp473dup | T3-7 |
| Délot, et al. Hum Mol Genet, 1999 | English | PSACH | 13 | c.1414_1419dup | p.Asp472_Asp473dup | T3-7 |
| Délot, et al. J Biol Chem, 1998 | English | PSACH | 13 | c.1414_1419dup | p.Asp472_Asp473dup | T3-7 |
| Jacob et al. Am J Med Genet A, 2022 | English | PSACH | 13 | c.1416_1421del | p.Asn474_Asp475del | T3-7 |
| Jacob et al. Am J Med Genet A, 2022 | English | PSACH | 13 | c.1417_1419del | p.Asp473del | T3-7 |
| Jacob et al. Am J Med Genet A, 2022 | English | PSACH | 13 | c.1417_1419del | p.Asp473del | T3-7 |
| Liang et al, Calcif Tissue Int, 2022 | English | PSACH | 13 | c.1417_1419del | p.Asp473del | T3-7 |
| Liang et al, Calcif Tissue Int, 2022 | English | PSACH | 13 | c.1417_1419del | p.Asp473del | T3-7 |
| Liang et al, Calcif Tissue Int, 2022 | English | PSACH | 13 | c.1417_1419del | p.Asp473del | T3-7 |
| Chen, et al. Front Endocrinol, 2022 | English | PSACH | 13 | c.1417_1419del | p.Asp473del | T3-7 |
| Kim, et al. Front Genet, 2021 | English | PSACH | 13 | c.1417_1419del | p.Asp473del | T3-7 |
| Yu, et al. Mol Med Rep, 2016 | English | PSACH | 13 | c.1417_1419del | p.Asp473del | T3-7 |
| Zhang, et al. J Hum Genet, 2015 | English | PSACH | 13 | c.1417_1419del | p.Asp473del | T3-7 |
| Briggs, et al. Eur J Hum Genet, 2014 | English | PSACH | 13 | c.1417_1419del | p.Asp473del | T3-7 |
| Briggs, et al. Eur J Hum Genet, 2014 | English | PSACH | 13 | c.1417_1419del | p.Asp473del | T3-7 |
| Briggs, et al. Eur J Hum Genet, 2014 | English | PSACH | 13 | c.1417_1419del | p.Asp473del | T3-7 |
| Briggs, et al. Eur J Hum Genet, 2014 | English | PSACH | 13 | c.1417_1419del | p.Asp473del | T3-7 |
| Briggs, et al. Eur J Hum Genet, 2014 | English | PSACH | 13 | c.1417_1419del | p.Asp473del | T3-7 |
| Jackson, et al. Hum Mutat, 2012 | English | PSACH | 13 | c.1417_1419del | p.Asp473del | T3-7 |
| Jackson, et al. Hum Mutat, 2012 | English | PSACH | 13 | c.1417_1419del | p.Asp473del | T3-7 |
| Jackson, et al. Hum Mutat, 2012 | English | PSACH | 13 | c.1417_1419del | p.Asp473del | T3-7 |
| Jackson, et al. Hum Mutat, 2012 | English | PSACH | 13 | c.1417_1419del | p.Asp473del | T3-7 |
| Jackson, et al. Hum Mutat, 2012 | English | PSACH | 13 | c.1417_1419del | p.Asp473del | T3-7 |
| Jung, et al. Int J Mol Med, 2010 | English | PSACH | 13 | c.1417_1419del | p.Asp473del | T3-7 |
| Jung, et al. Int J Mol Med, 2010 | English | PSACH | 13 | c.1417_1419del | p.Asp473del | T3-7 |
| Jung, et al. Int J Mol Med, 2010 | English | PSACH | 13 | c.1417_1419del | p.Asp473del | T3-7 |
| Kennedy, et al. Eur J Hum Genet, 2005 | English | PSACH | 13 | c.1417_1419del | p.Asp473del | T3-7 |
| Kennedy, et al. Eur J Hum Genet, 2005 | English | PSACH | 13 | c.1417_1419del | p.Asp473del | T3-7 |
| Kennedy, et al. Eur J Hum Genet, 2005 | English | PSACH | 13 | c.1417_1419del | p.Asp473del | T3-7 |
| Kennedy, et al. Eur J Hum Genet, 2005 | English | PSACH | 13 | c.1417_1419del | p.Asp473del | T3-7 |
| Kennedy, et al. Eur J Hum Genet, 2005 | English | PSACH | 13 | c.1417_1419del | p.Asp473del | T3-7 |
| Kennedy, et al. Eur J Hum Genet, 2005 | English | PSACH | 13 | c.1417_1419del | p.Asp473del | T3-7 |
| Kennedy, et al. Eur J Hum Genet, 2005 | English | PSACH | 13 | c.1417_1419del | p.Asp473del | T3-7 |
| Kennedy, et al. Eur J Hum Genet, 2005 | English | PSACH | 13 | c.1417_1419del | p.Asp473del | T3-7 |
| Kennedy, et al. Eur J Hum Genet, 2005 | English | PSACH | 13 | c.1417_1419del | p.Asp473del | T3-7 |
| Nakashima, et al. Am J Med Genet A, 2005 | English | PSACH | 13 | c.1417_1419del | p.Asp473del | T3-7 |
| Nakashima, et al. Am J Med Genet A, 2005 | English | PSACH | 13 | c.1417_1419del | p.Asp473del | T3-7 |
| Song, et al. J Pediatr Orthop B, 2004 | English | PSACH | 13 | c.1417_1419del | p.Asp473del | T3-7 |
| Song, et al. J Pediatr Orthop B, 2004 | English | PSACH | 13 | c.1417_1419del | p.Asp473del | T3-7 |
| Mabuchi, et al. Hum Genet, 2003 | English | PSACH | 13 | c.1417_1419del | p.Asp473del | T3-7 |
| Mabuchi, et al. Hum Genet, 2003 | English | PSACH | 13 | c.1417_1419del | p.Asp473del | T3-7 |
| Newman, et al. J Med Genet, 2000 | English | PSACH | 13 | c.1417_1419del | p.Asp473del | T3-7 |
| Loughlin, et al. Hum Mutat, 1998 | English | PSACH | 13 | c.1417_1419del | p.Asp473del | T3-7 |
| Loughlin, et al. Hum Mutat, 1998 | English | PSACH | 13 | c.1417_1419del | p.Asp473del | T3-7 |
| Ikegawa, et al. Hum Genet, 1998 | English | PSACH | 13 | c.1417_1419del | p.Asp473del | T3-7 |
| Ikegawa, et al. Hum Genet, 1998 | English | PSACH | 13 | c.1417_1419del | p.Asp473del | T3-7 |
| Ikegawa, et al. Hum Genet, 1998 | English | PSACH | 13 | c.1417_1419del | p.Asp473del | T3-7 |
| Deere, et al. Am J Med Genet, 1998 | English | PSACH | 13 | c.1417_1419del | p.Asp473del | T3-7 |
| Deere, et al. Am J Med Genet, 1998 | English | PSACH | 13 | c.1417_1419del | p.Asp473del | T3-7 |
| Hecht, et al. Matrix Biol, 1998 | English | PSACH | 13 | c.1417_1419del | p.Asp473del | T3-7 |
| Briggs, et al. Am J Hum Genet, 1998 | English | PSACH | 13 | c.1417_1419del | p.Asp473del | T3-7 |
| Briggs, et al. Am J Hum Genet, 1998 | English | PSACH | 13 | c.1417_1419del | p.Asp473del | T3-7 |
| Briggs, et al. Am J Hum Genet, 1998 | English | PSACH | 13 | c.1417_1419del | p.Asp473del | T3-7 |
| Briggs, et al. Am J Hum Genet, 1998 | English | PSACH | 13 | c.1417_1419del | p.Asp473del | T3-7 |
| Briggs, et al. Am J Hum Genet, 1998 | English | PSACH | 13 | c.1417_1419del | p.Asp473del | T3-7 |
| Briggs, et al. Am J Hum Genet, 1998 | English | PSACH | 13 | c.1417_1419del | p.Asp473del | T3-7 |
| Briggs, et al. Am J Hum Genet, 1998 | English | PSACH | 13 | c.1417_1419del | p.Asp473del | T3-7 |
| Cohn, et al. Ann N Y Acad Sci, 1996 | English | PSACH | 13 | c.1417_1419del | p.Asp473del | T3-7 |
| Cohn, et al. Ann N Y Acad Sci, 1996 | English | PSACH | 13 | c.1417_1419del | p.Asp473del | T3-7 |
| Cohn, et al. Ann N Y Acad Sci, 1996 | English | PSACH | 13 | c.1417_1419del | p.Asp473del | T3-7 |
| Cohn, et al. Ann N Y Acad Sci, 1996 | English | PSACH | 13 | c.1417_1419del | p.Asp473del | T3-7 |
| Cohn, et al. Ann N Y Acad Sci, 1996 | English | PSACH | 13 | c.1417_1419del | p.Asp473del | T3-7 |
| Hecht, et al. Nat Genet, 1995 | English | PSACH | 13 | c.1417_1419del | p.Asp473del | T3-7 |
| Hecht, et al. Nat Genet, 1995 | English | PSACH | 13 | c.1417_1419del | p.Asp473del | T3-7 |
| Hecht, et al. Nat Genet, 1995 | English | PSACH | 13 | c.1417_1419del | p.Asp473del | T3-7 |
| Hecht, et al. Nat Genet, 1995 | English | PSACH | 13 | c.1417_1419del | p.Asp473del | T3-7 |
| Hecht, et al. Nat Genet, 1995 | English | PSACH | 13 | c.1417_1419del | p.Asp473del | T3-7 |
| Xu, et al. Journal of Chongqing Medical University, 2022 | Chinese | PSACH | 13 | c.1417_1419del | p.Asp473del | T3-7 |
| Liang, et al. Chin J of Endocrinol Metab, 2019 | Chinese | PSACH | 13 | c.1417_1419del | p.Asp473del | T3-7 |
| Lv, et al. Chin J of Endocrinol Metab, 2019 | Chinese | PSACH | 13 | c.1417_1419del | p.Asp473del | T3-7 |
| Lv, et al. Chin J of Endocrinol Metab, 2019 | Chinese | PSACH | 13 | c.1417_1419del | p.Asp473del | T3-7 |
| Luo, et al. Chinese Journal of Reparative and Reconstructive Surgery, 2017 | Chinese | PSACH | 13 | c.1417_1419del | p.Asp473del | T3-7 |
| Zheng, et al. Chin J of Endocrinol Metab, 2016 | Chinese | PSACH | 13 | c.1417_1419del | p.Asp473del | T3-7 |
| Wang, et al. Shanghai Jiaotong University, 2014 | Chinese | PSACH | 13 | c.1417_1419del | p.Asp473del | T3-7 |
| Jackson, et al. Hum Mutat, 2012 | English | MED | 13 | c.1417_1419dup | p.Asp473dup | T3-7 |
| Jackson, et al. Hum Mutat, 2012 | English | MED | 13 | c.1417_1419dup | p.Asp473dup | T3-7 |
| Jackson, et al. Hum Mutat, 2012 | English | MED | 13 | c.1417_1419dup | p.Asp473dup | T3-7 |
| Délot, et al. Hum Mol Genet, 1999 | English | MED | 13 | c.1417_1419dup | p.Asp473dup | T3-7 |
| Délot, et al. J Biol Chem, 1998 | English | MED | 13 | c.1417_1419dup | p.Asp473dup | T3-7 |
| Cohn, et al. Ann N Y Acad Sci, 1996 | English | PSACH | 13 | c.1417_1419dup | p.Asp473dup | T3-7 |
| Yang, et al. Sun Yat-sen University, 2010 | Chinese | MED | 13 | c.1417_1419dup | p.Asp473dup | T3-7 |
| Vatanavicharn, et al. Am J Med Genet A, 2008 | English | PSACH | 13 | c.1417G>A | p.Asp473Asn | T3-7 |
| Deere, et al. Am J Med Genet, 1998 | English | PSACH | 13 | c.1417G>A | p.Asp473Asn | T3-7 |
| Kennedy, et al. Eur J Hum Genet, 2005 | English | PSACH | 13 | c.1417G>A | p.Asp473Asn | T3-7 |
| Jackson, et al. Hum Mutat, 2012 | English | PSACH | 13 | c.1417G>C | p.Asp473His | T3-7 |
| Zhang, et al. J Hum Genet, 2015 | English | PSACH | 13 | c.1417G>C | p.Asp473His | T3-7 |
| Song, et al. J Pediatr Orthop B, 2004 | English | PSACH | 13 | c.1417G>T | p.Asp473Tyr | T3-7 |
| Ikegawa, et al. Hum Genet, 1998 | English | PSACH | 13 | c.1418A>G | p.Asp473Gly | T3-7 |
| Jung, et al. Int J Mol Med, 2010 | English | PSACH | 13 | c.1418A>G | p.Asp473Gly | T3-7 |
| Tuncel, et al. Appl Immunohistochem Mol Morphol, 2021 | English | PSACH | 13 | c.1420_1422del | p.Asn474del | T3-7 |
| Briggs, et al. Eur J Hum Genet, 2014 | English | PSACH | 13 | c.1420_1425dup | p.Asn474_Asp475dup | T3-7 |
| Deere, et al. Am J Med Genet, 1998 | English | PSACH | 13 | c.1423G>A | p.Asp475Asn | T3-7 |
| Tariq, et al. Clin Genet, 208 | English | PSACH | 13 | c.1423G>A | p.Asp475Asn | T3-7 |
| Jackson, et al. Hum Mutat, 2012 | English | PSACH | 13 | c.1423G>A | p.Asp475Asn | T3-7 |
| Kennedy, et al. Eur J Hum Genet, 2005 | English | PSACH | 13 | c.1423G>A | p.Asp475Asn | T3-7 |
| Zhang, et al. J Hum Genet, 2015 | English | PSACH | 13 | c.1423G>C | p.Asp475His | T3-7 |
| Ichihashi, et al. Hum Genome Var, 2018 | English | PSACH | 13 | c.1426_1437del | p.Gly476_Asp479del | T3-7 |
| Kennedy, et al. Eur J Hum Genet, 2005 | English | MED | 13 | c.1435G>C | p.Asp479His | T3-7 |
| Kennedy, et al. Eur J Hum Genet, 2005 | English | PSACH | 13 | c.1435G>T | p.Asp479Tyr | T3-7 |
| Kim, et al. Am J Med Genet A, 2011 | English | MED | 13 | c.1444G>A | p.Asp482Asn | T3-7 |
| Kim, et al. Am J Med Genet A, 2011 | English | MED | 13 | c.1444G>A | p.Asp482Asn | T3-7 |
| Jung, et al. Int J Mol Med, 2010 | English | PSACH | 13 | c.1444G>A | p.Asp482Asn | T3-7 |
| Song, et al. J Pediatr Orthop B, 2004 | English | PSACH | 13 | c.1444G>C | p.Asp482His | T3-7 |
| Yang, et al. Xi’an Medical University, 2024 | Chinese | MED | 13 | c.1444G>T | p.Asp482Tyr | T3-7 |
| Jackson, et al. Hum Mutat, 2012 | English | PSACH | 13 | c.1445A>G | p.Asp482Gly | T3-7 |
| Susic, et al. Hum Mutat, 1998 | English | PSACH | 13 | c.1445A>G | p.Asp482Gly | T3-7 |
| Jacob et al. Am J Med Genet A, 2022 | English | PSACH | 13 | c.1445A>T | p.Asp482Val | T3-7 |
| Donkervoort, et al. Neuromuscul Disord, 2013 | English | PSACH | 13 | c.1450T>G | p.Cys484Gly | T3-7 |
| Mabuchi, et al. Hum Genet, 2003 | English | PSACH | 13 | c.1450T>G | p.Cys484Gly | T3-7 |
| Sakamoto, et al. J Orthop Sci, 2017 | English | MED | 13 | c.1467C>A | p.Asn489Lys | T3-7 |
| Briggs, et al. Eur J Hum Genet, 2014 | English | MED | 13 | c.1467C>A | p.Asn489Lys | T3-7 |
| Kennedy, et al. Eur J Hum Genet, 2005 | English | MED | 13 | c.1475A>C | p.Gln492Pro | T3-7 |
| Mabuchi, et al. Hum Genet, 2003 | English | MED | 14 | c.1501_1509del | p.Gly501_Val503del | T3-8 |
| Jackson, et al. Hum Mutat, 2012 | English | MED | 14 | c.1502G>A | p.Gly501Asp | T3-8 |
| Jackson, et al. Hum Mutat, 2012 | English | MED | 14 | c.1502G>A | p.Gly501Asp | T3-8 |
| Jackson, et al. Hum Mutat, 2012 | English | MED | 14 | c.1502G>A | p.Gly501Asp | T3-8 |
| Jackson, et al. Hum Mutat, 2012 | English | MED | 14 | c.1502G>A | p.Gly501Asp | T3-8 |
| Briggs, et al. Eur J Hum Genet, 2014 | English | PSACH | 14 | c.1510T>C | p.Cys504Arg | T3-8 |
| Jacob et al. Am J Med Genet A, 2022 | English | PSACH | 14 | c.1511G>A | p.Cys504Tyr | T3-8 |
| Xie, et al. Gene, 2013 | English | PSACH | 14 | c.1511G>A | p.Cys504Tyr | T3-8 |
| Kennedy, et al. Eur J Hum Genet, 2005 | English | PSACH | 14 | c.1511G>C | p.Cys504Ser | T3-8 |
| Yu, et al. Mol Med Rep, 2016 | English | PSACH | 14 | c.1512C>G | p.Cys504Trp | T3-8 |
| Lv, et al. Chin J of Endocrinol Metab, 2019 | Chinese | PSACH | 14 | c.1512C>G | p.Cys504Trp | T3-8 |
| Kim, et al. Am J Med Genet A, 2011 | English | MED | 14 | c.1519G>A | p.Asp507Asn | T3-8 |
| Kim, et al. Front Genet, 2021 | English | MED | 14 | c.1519G>A | p.Asp507Asn | T3-8 |
| Hasegawa, et al. Clin Pediatr Endocrinol, 2023 | English | PSACH | 14 | c.1519G>T | p.Asp507Tyr | T3-8 |
| Jackson, et al. Hum Mutat, 2012 | English | PSACH | 14 | c.1520A>G | p.Asp507Gly | T3-8 |
| Deere, et al. Am J Med Genet, 1998 | English | PSACH | 14 | c.1520A>G | p.Asp507Gly | T3-8 |
| Jung, et al. Int J Mol Med, 2010 | English | PSACH | 14 | c.1525G>A | p.Asp509Asn | T3-8 |
| Deere, et al. Am J Med Genet, 1998 | English | PSACH | 14 | c.1526A>C | p.Asp509Ala | T3-8 |
| Zhang, et al. J Hum Genet, 2015 | English | PSACH | 14 | c.1526A>G | p.Asp509Gly | T3-8 |
| Zhang, et al. J Hum Genet, 2015 | English | PSACH | 14 | c.1526A>G | p.Asp509Gly | T3-8 |
| Kennedy, et al. Eur J Hum Genet, 2005 | English | PSACH | 14 | c.1526A>G | p.Asp509Gly | T3-8 |
| Deere, et al. Am J Med Genet, 1998 | English | PSACH | 14 | c.1526A>G | p.Asp509Gly | T3-8 |
| Liang et al, Calcif Tissue Int, 2022 | English | PSACH | 14 | c.1526A>T | p.Asp509Val | T3-8 |
| Zhang, et al. J Hum Genet, 2015 | English | PSACH | 14 | c.1526A>T | p.Asp509Val | T3-8 |
| Mabuchi, et al. Hum Genet, 2003 | English | PSACH | 14 | c.1527T>G | p.Asp509Glu | T3-8 |
| Kennedy, et al. Eur J Hum Genet, 2005 | English | PSACH | 14 | c.1528_1539del | p.Ala510_Val513del | T3-8 |
| Liang et al, Calcif Tissue Int, 2022 | English | PSACH | 14 | c.1531G>A | p.Asp511Asn | T3-8 |
| Deere, et al. Am J Med Genet, 1998 | English | PSACH | 14 | c.1531G>C | p.Asp511His | T3-8 |
| Briggs, et al. Eur J Hum Genet, 2014 | English | PSACH | 14 | c.1531G>T | p.Asp511Tyr | T3-8 |
| Hecht, et al. J Orthop Res, 2004 | English | PSACH | 13 | c.1531G>T | P.Asp511Tyr | T3-8 |
| Tufan, et al. Eur J Hum Genet, 2007 | English | PSACH | 14 | c.1532A>G | p.Asp511Gly | T3-8 |
| Jackson, et al. Hum Mutat, 2012 | English | PSACH | 14 | c.1532A>G | p.Asp511Gly | T3-8 |
| Briggs, et al. Eur J Hum Genet, 2014 | English | PSACH | 14 | c.1533C>G | p.Asp511Glu | T3-8 |
| Susic, et al. Clin Genet, 1997 | English | PSACH | 14 | c.1537_1548del | p.Val513_Lys516del | T3-8 |
| Lin, et al. Pediatr Neonatol, 2018 | English | MED | 14 | c.1538T>G | p.Val513Gly | T3-8 |
| Vranka, et al. Matrix Biol, 2001 | English | PSACH | 14 | c.1544A>G | p.Asp515Gly | T3-8 |
| Jackson, et al. Hum Mutat, 2012 | English | PSACH | 14 | c.1544A>G | p.Asp515Gly | T3-8 |
| Liang et al, Calcif Tissue Int, 2022 | English | PSACH | 14 | c.1552G>A | p.Asp518Asn | T3-8 |
| Chen, et al. Front Endocrinol, 2022 | English | PSACH | 14 | c.1552G>A | p.Asp518Asn | T3-8 |
| Ikegawa, et al. Hum Genet, 1998 | English | PSACH | 14 | c.1552G>A | p.Asp518Asn | T3-8 |
| Liang, et al. Chin J of Endocrinol Metab, 2019 | Chinese | PSACH | 14 | c.1552G>A | p.Asp518Asn | T3-8 |
| Kennedy, et al. Eur J Hum Genet, 2005 | English | PSACH | 14 | c.1552G>A | p.Asp518Asn | T3-8 |
| Deere, et al. Am J Med Genet, 1998 | English | PSACH | 14 | c.1552G>A | p.Asp518Asn | T3-8 |
| Deere, et al. Am J Med Genet, 1999 | English | PSACH | 14 | c.1552G>C | p.Asp518His | T3-8 |
| Xia, et al. Chin Med J (Engl), 2013 | English | MED | 14 | c.1552G>T | p.Asp518Tyr | T3-8 |
| Liu, et al. Peking Union Medical College, 2010 | Chinese | MED | 14 | c.1552G>T | p.Asp518Tyr | T3-8 |
| Kennedy, et al. Eur J Hum Genet, 2005 | English | PSACH | 14 | c.1553A>G | p.Asp518Gly | T3-8 |
| Hasegawa, et al. Clin Pediatr Endocrinol, 2023 | English | PSACH | 14 | c.1553A>T | p.Asp518Val | T3-8 |
| Singh, et al. J Clin Diagn Res, 2013 | English | PSACH | 14 | c.1554C>G | p.Asp518Glu | T3-8 |
| Ballo, et al. Am J Med Genet, 1997 | English | MED | 14 | c.1569C>G | p.Asn523Lys | T3-8 |
| Jackson, et al. Hum Mutat, 2012 | English | MED | 14 | c.1569C>G | p.Asn523Lys | T3-8 |
| Jackson, et al. Hum Mutat, 2012 | English | MED | 14 | c.1569C>G | p.Asn523Lys | T3-8 |
| Jackson, et al. Hum Mutat, 2012 | English | MED | 14 | c.1569C>G | p.Asn523Lys | T3-8 |
| Liang et al, Calcif Tissue Int, 2022 | English | PSACH | 14 | c.1576G>T | p.Val526Phe | T3-8 |
| Hecht, et al. Matrix Biol, 1998 | English | PSACH | 14 | c.1579A>G | p.Thr527Ala | T3-8 |
| Liang et al, Calcif Tissue Int, 2022 | English | PSACH | 14 | c.1585A>G | p.Thr529Ala | CTD |
| Yu, et al. Mol Med Rep, 2016 | English | PSACH | 14 | c.1585A>G | p.Thr529Ala | CTD |
| Lv, et al. Chin J of Endocrinol Metab, 2019 | Chinese | PSACH | 14 | c.1585A>G | p.Thr529Ala | CTD |
| Zhang, et al. J Hum Genet, 2015 | English | PSACH | 14 | c.1585A>G | p.Thr529Ala | CTD |
| Mazzotti, et al. Genes, 2024 | English | MED | 14 | c.1586C>A | p.Thr529Asn | CTD |
| Jackson, et al. Hum Mutat, 2012 | English | PSACH | 14 | c.1586C>T | p.Thr529Ile | CTD |
| Jackson, et al. Hum Mutat, 2012 | English | PSACH | 14 | c.1586C>T | p.Thr529Ile | CTD |
| Kennedy, et al. Hum Mutat, 2005 | English | PSACH | 14 | c.1586C>T | p.Thr529Ile | CTD |
| Kennedy, et al. Eur J Hum Genet, 2005 | English | PSACH | 14 | c.1586C>T | p.Thr529Ile | CTD |
| Kennedy, et al. Hum Mutat, 2005 | English | MED | 14 | c.1665C>A | p.Asn555Lys | CTD |
| Kennedy, et al. Eur J Hum Genet, 2005 | English | MED | 14 | c.1665C>A | p.Asn555Lys | CTD |
| Kennedy, et al. Hum Mutat, 2005 | English | MED | 14 | c.1665C>G | p.Asn555Lys | CTD |
| Kennedy, et al. Eur J Hum Genet, 2005 | English | MED | 14 | c.1665C>G | p.Asn555Lys | CTD |
| Gu, et al. Biomed Res Int, 2017 | English | PSACH | 15 | c.1675G>A | p.Glu559Lys | CTD |
| Qiu, et al. China Medical University, 2020 | Chinese | PSACH | 15 | c.1675G>A | p.Glu559Lys | CTD |
| Qiu, et al. Mutat Res, 2022 | English | PSACH | 15 | c.1675G>A | p.Glu559Lys | CTD |
| Deere, et al. Am J Med Genet, 1999 | English | PSACH | 16 | c.1747G>A | p.Glu583Lys | CTD |
| Briggs, et al. Eur J Hum Genet, 2014 | English | MED | 16 | c.1754C>A | p.Thr585Lys | CTD |
| Kennedy, et al. Hum Mutat, 2005 | English | PSACH | 16 | c.1754C>G | p.Thr585Arg | CTD |
| Jackson, et al. Hum Mutat, 2012 | English | PSACH | 16 | c.1754C>G | p.Thr585Arg | CTD |
| Briggs, et al. Am J Hum Genet, 1998 | English | MED | 16 | c.1754C>G | p.Thr585Arg | CTD |
| Jackson, et al. Hum Mutat, 2012 | English | MED | 16 | c.1754C>T | p.Thr585Met | CTD |
| Briggs, et al. Am J Hum Genet, 1998 | English | PSACH | 16 | c.1754C>T | p.Thr585Met | CTD |
| Song, et al. J Hum Genet, 2003 | English | MED | 16 | c.1754C>T | p.Thr585Met | CTD |
| Deere, et al. Am J Med Genet, 1998 | English | PSACH | 16 | c.1760A>G | p.His587Arg | CTD |
| Song, et al. J Pediatr Orthop B, 2004 | English | PSACH | 16 | c.1760A>G | p.His587Arg | CTD |
| Kennedy, et al. Hum Mutat, 2005 | English | MED | 16 | c.1813G>A | p.Asp605Asn | CTD |
| Kennedy, et al. Eur J Hum Genet, 2005 | English | MED | 16 | c.1813G>C | p.Asp605Asn | CTD |
| Liu, et al. Journal of Tianjin Medical University, 2022 | Chinese | PSACH | 17 | c.2041T>A | p.Ser681Thr | CTD |
| Kennedy, et al. Hum Mutat, 2005 | English | MED | 17 | c.2042C>G | p.Ser681Cys | CTD |
| Briggs, et al. Eur J Hum Genet, 2014 | English | MED | 18 | c.2152C>T | p.Arg718Trp | CTD |
| Kim, et al. Am J Med Genet A, 2011 | English | MED | 18 | c.2152C>T | p.Arg718Trp | CTD |
| Kim, et al. Am J Med Genet A, 2011 | English | MED | 18 | c.2152C>T | p.Arg718Trp | CTD |
| Kennedy, et al. Hum Mutat, 2005 | English | MED | 18 | c.2152C>T | p.Arg718Trp | CTD |
| Jakkula, et al. J Med Genet, 2003 | English | MED | 18 | c.2152C>T | p.Arg718Trp | CTD |
| Jakkula, et al. J Med Genet, 2003 | English | MED | 18 | c.2152C>T | p.Arg718Trp | CTD |
| Mabuchi, et al. Hum Genet, 2003 | English | MED | 18 | c.2152C>T | p.Arg718Trp | CTD |
| Jackson, et al. Hum Mutat, 2012 | English | MED | 18 | c.2152C>T | p.Arg718Trp | CTD |
| Jackson, et al. Hum Mutat, 2012 | English | MED | 18 | c.2152C>T | p.Arg718Trp | CTD |
| Kennedy, et al. Hum Mutat, 2005 | English | MED | 18 | c.2152C>T | p.Arg718Trp | CTD |
| Hsu, et al. Connect Tissue Res, 2021 | English | MED？ | 18 | c.2152C>T | p.Arg718Trp | CTD |
| Jung, et al. Int J Mol Med, 2010 | English | PSACH | 18 | c.2152C>T | p.Arg718Trp | CTD |
| López, et al. Mol Syndromol, 2023 | English | PSACH | 18 | c.2153G>C | p.Arg718Pro | CTD |
| Jackson, et al. Hum Mutat, 2012 | English | MED | 18 | c.2153G>C | p.Arg718Pro | CTD |
| Kennedy, et al. Hum Mutat, 2005 | English | MED | 18 | c.2153G>C | p.Arp718Pro | CTD |
| Kennedy, et al. Eur J Hum Genet, 2005 | English | MED | 18 | c.2153G>C | p.Arg718Pro | CTD |
| Kennedy, et al. Hum Mutat, 2005 | English | PSACH | 18 | c.2155G>A | p.Gly719Ser | CTD |
| Jackson, et al. Hum Mutat, 2012 | English | PSACH | 18 | c.2155G>A | p.Gly719Ser | CTD |
| Kennedy, et al. Eur J Hum Genet, 2005 | English | PSACH | 18 | c.2155G>A | p.Gly719Ser | CTD |
| Mabuchi, et al. Am J Med Genet, 2001 | English | PSACH | 18 | c.2156G>A | p.Gly719Asp | CTD |
| Li, et al. Chin J Contemp Pediatr, 2013 | Chinese | PSACH | 18 | c.2156G>A | p.Gly719Asp | CTD |
| Jakkula, et al. Eur J Hum Genet, 2005 | English | MED | 18 | c.2204C>T | p.Ala735Val | CTD |
| Mabuchi, et al. Hum Genet, 2003 | English | MED | 18 | c.2223_2224insC | p.Asn742Glnfs*2 | CTD |
