# Supplementary material for: Genotype-phenotype correlations in PSACH/EDM1 patients with COMP gene variants: a comprehensive review of 830 cases
Source: Front Endocrinol (Lausanne). 2026 Feb 19;17:1740770. doi: 10.3389/fendo.2026.1740770 (PMC12960193; doi:10.3389/fendo.2026.1740770)
Supplement: Supplementary file 3 [file Table3.docx]

**Figure legend**

**Supplemental Figure 1**. Distribution of Z-score of height and onset age in patients with PSACH (n = 64) and EDM1 (n = 37). PSACH, pseudoachondroplasia; EDM1, multiple epiphyseal dysplasia-1.
