# Supplementary figures and images for: Genotype-phenotype correlations in PSACH/EDM1 patients with COMP gene variants: a comprehensive review of 830 cases
Source: Front Endocrinol (Lausanne). 2026 Feb 19;17:1740770. doi: 10.3389/fendo.2026.1740770 (PMC12960193; doi:10.3389/fendo.2026.1740770)

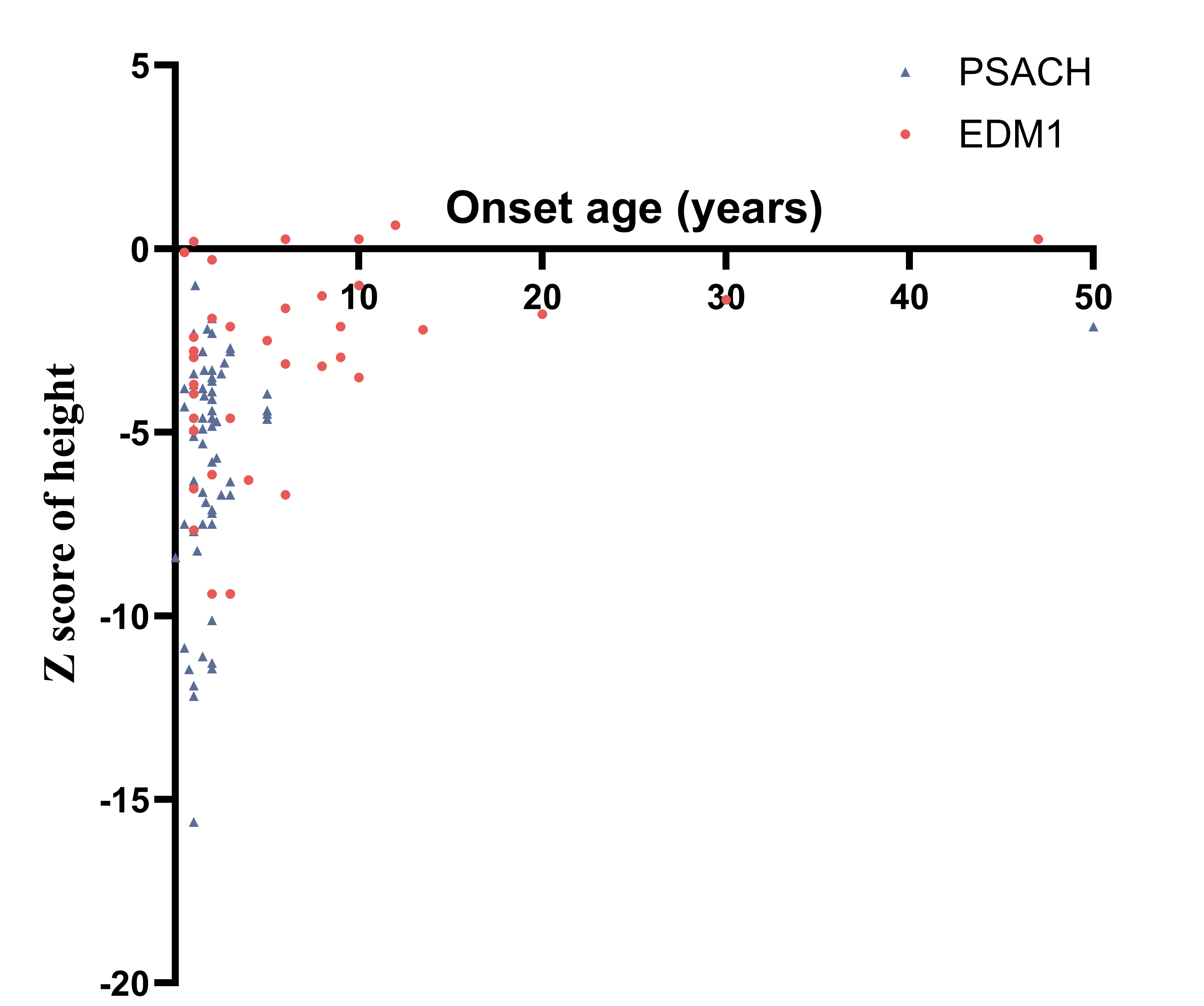

Supplement: Supplementary file 4 [file Image1.tif]
